# Efficient labeling and imaging of protein-coding genes in living cells using CRISPR-Tag

**DOI:** 10.1038/s41467-018-07498-y

**Published:** 2018-11-29

**Authors:** Baohui Chen, Wei Zou, Haiyue Xu, Ying Liang, Bo Huang

**Affiliations:** 1 Department of Cell Biology, and Bone Marrow Transplantation Center of the First Affiliated Hospital, Zhejiang University School of Medicine, Zhejiang, Hangzhou, 310058 China; 2 Institute of Hematology, Zhejiang University & Zhejiang Engineering Laboratory for Stem Cell and Immunotherapy, Hangzhou, 310003 China; 3 The Fourth Affiliated Hospital, Zhejiang University School of Medicine, Yiwu, 322000 China; 4 Insititute of Translational Medicine, Zhejiang University, Hangzhou, 310058 China; 5 Department of Pharmaceutical Chemistry, University of California, San Francisco, San Francisco, 94143 CA USA; 6 Department of Biochemistry and Biophysics, University of California, San Francisco, San Francisco, 94143 CA USA; 7 Chan Zuckerberg Biohub, San Francisco, 94158 CA USA

## Abstract

The lack of efficient tools to image non-repetitive genes in living cells has limited our ability to explore the functional impact of the spatiotemporal dynamics of such genes. Here, we addressed this issue by developing a CRISPR-Tag system using one to four highly active sgRNAs to specifically label protein-coding genes with a high signal-to-noise ratio for visualization by wide-field fluorescence microscopy. Our approach involves assembling a CRISPR-Tag within the intron region of a fluorescent protein and then integrating this cassette to N- or C-terminus of a specific gene, which enables simultaneous real-time imaging of protein and DNA of human protein-coding genes, such as HIST2H2BE, LMNA and HSPA8 in living cells. This CRISPR-Tag system, with a minimal size of ~250 bp DNA tag, represents an easily and broadly applicable technique to study the spatiotemporal organization of genomic elements in living cells.

## Abstract (web summary)

Difficulties in imaging non-repetitive genes impair our ability to explore their dynamics. Here the authors present CRISPR-Tag, which marks genes of interest with small repeat sequences to recruit dCas9-GFP.

## Introduction

Individual genes and genomic regions are located at different positions in the three-dimensional space of the nucleus^1,2^. The long-standing questions are whether the position of a gene affects its activity and how the gene positioning is maintained and regulated. There is no doubt that utilizing imaging techniques, which allow direct visualization of gene positioning and gene expression in living cells simultaneously, we will be able to uncover how gene position is linked to gene activity. Recent efforts toward this end focused on engineering a series of modular proteins with specific DNA recognition, including the clustered regularly interspaced short palindromic repeat (CRISPR)-CRISPR-associated (Cas) system^3–5^. The catalytically dead version of Cas9 (dCas9) has been extensively explored for imaging endogenous genomic loci in living cells^6,7^. However, most of targets visualized by dCas9 system are still limited to repetitive genomic region.

The major challenge is, when targeting non-repetitive genomic regions, it requires multiple sgRNAs function simultaneously to provide a sufficient signal-to-noise ratio for microscopy detection^6^. For example, to visualize a non-repetitive gene or regulatory element in mouse embryonic stem cells, 36 sgRNAs were expressed from three CARGO arrays to achieve efficient labeling^8^. Although two groups reported that the number of sgRNAs could be reduced to 3–4 using a combination of signal amplification and super-resolution microscopy^9,10^, the labeling efficacy has not been quantitatively assessed. It is worth noting that signal amplification using multiple MS2 or PP7 repeats may introduce unspecific spots due to accumulation of nascent tagged sgRNA transcripts^11^.

It is a general issue for all CRISPR applications that the efficiency of Cas9 targeting for any genomic locus can be dramatically influenced by the efficiency of sgRNAs used^12^. As such, it is very likely that only part of sgRNAs selected for DNA labeling function with high efficiency, which remains the major uncertainty of CRISPR-mediated genomic labeling. Thus, well-designed approaches using CRISPR imaging as readouts are critical to further optimize the DNA labeling system. Collectively, it is vital to achieve full potential of CRISPR imaging technology for labeling non-repetitive genomic elements. As such, we aim to develop DNA tags consisted of DNA sequence, which can be efficiently bound by dCas9-FP with highly active sgRNAs. In fluorescent repressor operator system (FROS), repeating sequences of Lac operator (LacO, 256 repeats) or Tet operator (TetO, 96 repeats) are used as DNA tags. Due to the large size and highly repetitive nature of LacO/TetO array (usually ~10 and ~4 kb, respectively)^13,14^, it remains technically challenging to use LacO/TetO DNA tags to label a specific endogenous gene. Different from FROS system, DNA sequence recognized by dCas9-FP is simply restricted by “NGG” PAM sequence. Therefore, we sought to assemble a shorter and more versatile DNA tag based on the CRISPR-Cas9 systems.

Here, we developed another type of DNA tags, termed “CRISPR-Tag”, to label endogenous protein-coding genes in living cells. Two to six repeats of CRISPR targetable DNA sequences from *Caenorhabditis elegans* genome were integrated to a specific human gene and then imaged by fluorescent dCas9. We demonstrated a DNA tag knock-in strategy which allows simultaneous imaging of DNA and protein of the target gene. Most importantly, the use of CRISPR-Tags does not affect gene expression. All CRISPR-Tags we created are smaller than 850 bp. Thus, the CRISPR-Tag system represents an efficient, easy, and scalable DNA tagging system in living human cells.

## Results

### Assembly of CRISPR-Tag for labeling non-repetitive genes

**Fig. 1:**
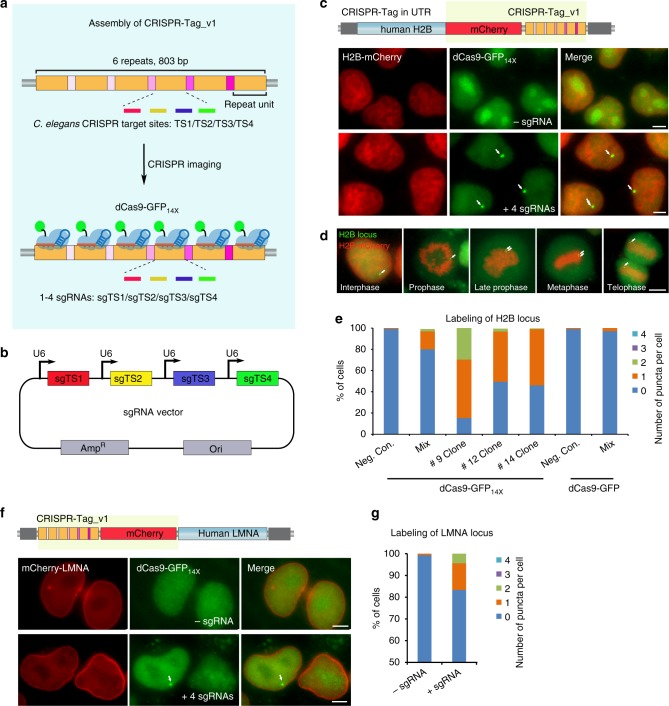
Development of CRISPR-Tag to label non-repetitive genes. **a** Schematic of CRISPR-Tag design as a DNA tagging system. **b** Co-expression of four sgRNAs in one vector. sgTS1, sgTS2, sgTS3, and sgTS4 were built individually and then sub-cloned into a single vector. **c** mCherry-CRISPR-Tag_v1 was inserted into C-terminus of human H2B gene as highlighted by yellow. Representative images denoted simultaneous visualization of H2B-mCherry and H2B loci by using CRISPR-Tag. H2B loci were labeled by dCas9-GFP_14X_ with four sgRNAs (sgTS1–sgTS4). **d** H2B-mCherry and H2B loci were visualized at different stages of cell cycles. **e** Quantifications of H2B locus labeling efficiency, *n* ≥ 84 cells. **f** Representative images to show simultaneous visualization of mCherry-LMNA and LMNA loci by utilizing the CRISPR-Tag system. **g** Quantifications of LMNA locus labeling efficiency, *n* ≥ 150 cells. All images in **c**, **d**, and **f** are maximum intensity projections from z stacks. Scale bars: 5 µm

CRISPR-Tag (diagram in Fig. 1a) was assembled with CRISPR-targeting sequences from *C. elegans* genome, which have been characterized for genome editing by several studies^15–18^. Six target sequences were picked according to the editing efficiency in worms and the on/off-target activity prediction by the web tool (http://crispr.mit.edu/). In addition, we generated a piece of artificial sequence based on the preference of nucleotides sequences that impact sgRNA efficacy^19^. In total, seven sgRNAs, termed sgTS1–sgTS7, were selected as the candidate sequences to assemble CRISPR-Tags (Supplementary Table 1). The first version of CRISPR-Tag (CRISPR-Tag_v1) contains six repeats. Four CRISPR-targeting sequences, TS1–TS4 were arranged in each repeat unit. Six repeat units were ligated to form a CRISPR-Tag using Golden Gate assembly. There are unique spacer sequences (25 bp) in between the repeats, which allows PCR or DNA sequencing to validate CRISPR-Tag sequence in cloning and knock-in experiments (Supplementary Fig. 1). To label a specific non-repetitive gene, we aim to first insert CRISPR-Tag into its 3′ UTR region or intron region by CRISPR knock-in, and then label the CRISPR-Tag with the nuclease-deficient Cas9 (dCas9) fused with fluorescent tags (Fig. 1a).

### Signal amplification using tandem split GFP system

In order to minimize the size of the CRISPR-Tag, i.e. the number of target sequences, required to generate detectable signal, we adopted the tandem split GFP system to amplify the fluorescence from dCas9^20^. To this end, we fused a repeating array containing 14 copies of GFP11 tags to dCas9 (dCas9-GFP_14x_), which can theoretically recruit as many as 14 copies of GFP (Supplementary Fig. 2a). When labeling the repeats in *MUC4* and 5S rDNA genes and imaging with wide-field fluorescence microscopy, dCas9-GFP_14x_ increased the signal-to-noise ratio (SNR) by a factor of 3 compared to dCas9-EGFP (Supplementary Fig. 2b, c), demonstrating an enhanced microscopy detection efficiency. However, when imaging non-repetitive regions in *MUC4* gene with our previously published 36 sgRNAs that have not been individually validated, signal amplification alone by dCas9-GFP_14x_ still gives relatively low SNR. Only ~26% of cells contain GFP-labeled spots due to low SNR (Supplementary Fig. 3). This result further indicates the need for a small set of well-validated sgRNAs as in our CRIPSR-Tag system.

### CRISPR-Tag enables both DNA and protein labeling of genes

To demonstrate our CRISPR-Tag, we chose a protein-coding gene as our test system in order to simplify the selection process of CRISPR-Tag knock-in cells. Specifically, we inserted CRISPR-Tag into the 3′ UTR region of human HIST2H2BE (encoding histone H2B) by CRISPR knock-in using electroporation of Cas9/sgRNA ribonucleoprotein complexes (RNPs)^21^ and double-cut plasmid donor^22^, which could increase targeting specificity and efficiency, respectively (diagram in Supplementary Fig. 4). We found that double-cut plasmid donor indeed resulted in higher knock-in efficiency than conventional plasmid donor and the efficiency highly dependents on cell types (Supplementary Fig. 5a, b). To select the integrated cells, we further added a mCherry sequence to the tag, which was knocked into the C-terminus of H2B as a FACS sorting marker (Supplementary Fig. 5c). Although most knock-in efficiencies were lower than 1%, positive cells were successfully isolated by FACS sorting to generate stable CRISPR-Tag knock-in cell lines (Supplementary Table 2). CRISPR-Tag insertion was then validated by PCR and was further confirmed by the subcellular localization of H2B-mCherry (Supplementary Figs. 5e and 6).

We then performed CRISPR imaging experiments using dCas9-GFP_14x_ Tag. As expected, one bright puncta, representing H2B locus, was clearly visible upon transfection of four sgRNAs (sgTS1–sgTS4) (Fig. 1b, c). Histone H2B, as one of the five main histone proteins, is involved in the formation of chromatin structure in eukaryotic cells^23^. H2B-mCherry allowed imaging of both interphase chromatin and mitotic chromosomes and revealed various chromatin states. In the meanwhile, we observed that H2B locus underwent replication and separation into two daughter cells at the telophase stage (Fig. 1d**;** Supplementary Movie 1). We observed that ~17% of H2B-mCherry^+^ mix pool cells showed a single, clearly GFP-tagged spot, indicating only one of the alleles was successfully modified by CRISPR editing. ~2% of cells have two spots, which might be due to DNA replication during the cell cycle or modifications of both alleles. The other 80% of cells did not display clear fluorescent spots, which might be attributed to sub-optimal expression of dCas9-GFP11_14x_, GFP1-10, and/or sgRNA in this mixed pool. We isolated three single cell clones which showed specific labeling (Clones 9, 12, and 14). All three had high labeling efficiency, 85%, 51%, and 54%, respectively (Fig. 1e**;** Supplementary Fig. 7). Nearly 100% of labeled cells in Clone 12 and 14 only contained a single spot, while about 30% of labeled cells in Clone 9 showed two spots. The difference is likely due to the number of modified alleles (Supplementary Fig. 6e). Taken together, we suggest that clonal isolation is ideal for increasing labeling efficiency, but is not necessary.

Next, we asked if signal amplification using tandem split GFP is a must for CRISPR-Tag system. To address this question, we carried out CRISPR-Tag knock-in to label H2B locus in dCas9-EGFP cells. No clearly visible spots were observed, suggesting that signal amplification using dCas9-GFP_14X_ is critical in this DNA tagging system (Fig. 1e). Similarly, we achieved simultaneous imaging of protein and gene positioning for both human nuclear membrane protein LMNA, and heat-shock response protein HSPA8 in living cells by CRISPR-Tag system (Fig. 1f, g**;** Supplementary Figs. 5d, f and 8).

### CRISPR-Tag in introns minimally affects gene expression

**Fig. 2:**
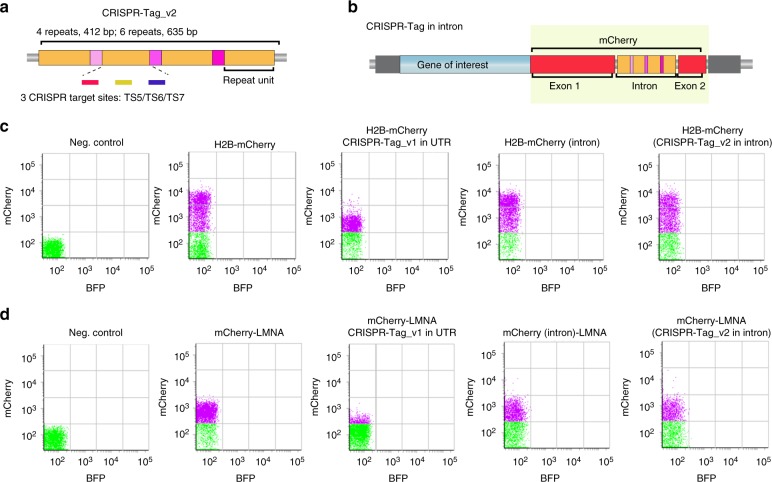
The protein expression remains normal when hiding CRISPR-Tag in intron. **a** Schematic of CRISPR-Tag_v2 design which contains four repeats. Each repeat harbors three CRISPR-targeting sites including TS5, TS6, and TS7. **b** Schematic of CRISPR-Tag positioning in the intron of mCherry. The fragment including mCherry and CRISPR-Tag, indicated by yellow, was integrated to the N-terminus or C-terminus of a target gene. **c**, **d** FACS analysis was performed to characterize H2B-mCherry (**c**) or mCherry-LMNA (**d**) expression in HeLa cells. Each dot represents single cells. BFP serves as an irrelevant channel. The distribution of dots along the *y*-axis indicates mCherry signal intensity

Specifically when tagging protein-coding genes, it is possible that certain 3′ UTR sequences may affect the expression level. Indeed, comparing to inserting mCherry alone, inserting mCherry together with CRISPR-Tag caused a decrease in protein expression level for H2B, LMNA, and HSPA8 in HeLa cells, but not H2B in 293T cells (Fig. 2; Supplementary Fig. 9). To minimalize this effect, we engineered the mCherry coding sequence to add in an intron while maintaining its expression level (Supplementary Fig. 10a, b). We have also assembled a second version of CRISPR-Tag, termed CRISPR-Tag_v2, which can be recognized by sgTS5, sgTS6, and sgTS7. We designed two different lengths of CRISPR-Tag_v2, containing 4 and 6 repeats, respectively (Fig. 2a, b). When inserting in the mCherry intron, CRISPR-Tag_v2 had no effect on H2B-mCherry expression, whereas our earlier design CRISPR-Tag_v1 caused a decrease in mCherry expression, possibly due to its interference with splicing (Supplementary Figs. 10c, d).

Then, we validated CRISPR-Tag for the labeling and imaging of H2B and LMNA genes. With this strategy, both H2B and LMNA genes could be efficiently labeled using three sgRNAs while not affecting their expression levels (Fig. 2c, d**;** Supplementary Fig. 11). To further confirm whether CRISPR-Tag insertions disrupt gene transcription, we compared the transcription level of the fluorescent fusion of H2B and LMNA with or without CRISPR-Tag insertions. Relative RNA expression was normalized against endogenous genes (Supplementary Fig. 12). We did not observe significant differences between the control and samples, indicating that our strategy generates no obvious disruption to transcription. It is worth noting that quantitative PCR results indicated that CRISPR-Tag in UTR region has little effect on transcription, which is probably due to the limit of qPCR detection. FACS analysis directly quantifies protein expression level of CRISR-Tag knock-in allele, while qPCR measures transcription of all alleles in the cell.

Because dCas9-GFP_14X_ would be much larger than a single GFP tag, we tested whether the labeling of protein-coding genes by dCas9-GFP_14X_ could affect protein expression. We compared the expression of H2B-mCherry in cells with or without CRISPR-positive signal and found no significant difference (Supplementary Fig. 13). Together, these results suggest that the CRISPR-Tag system does not obviously perturb gene expression.

### CRISPR-Tag allows long-term tracking of DNA and protein

**Fig. 3:**
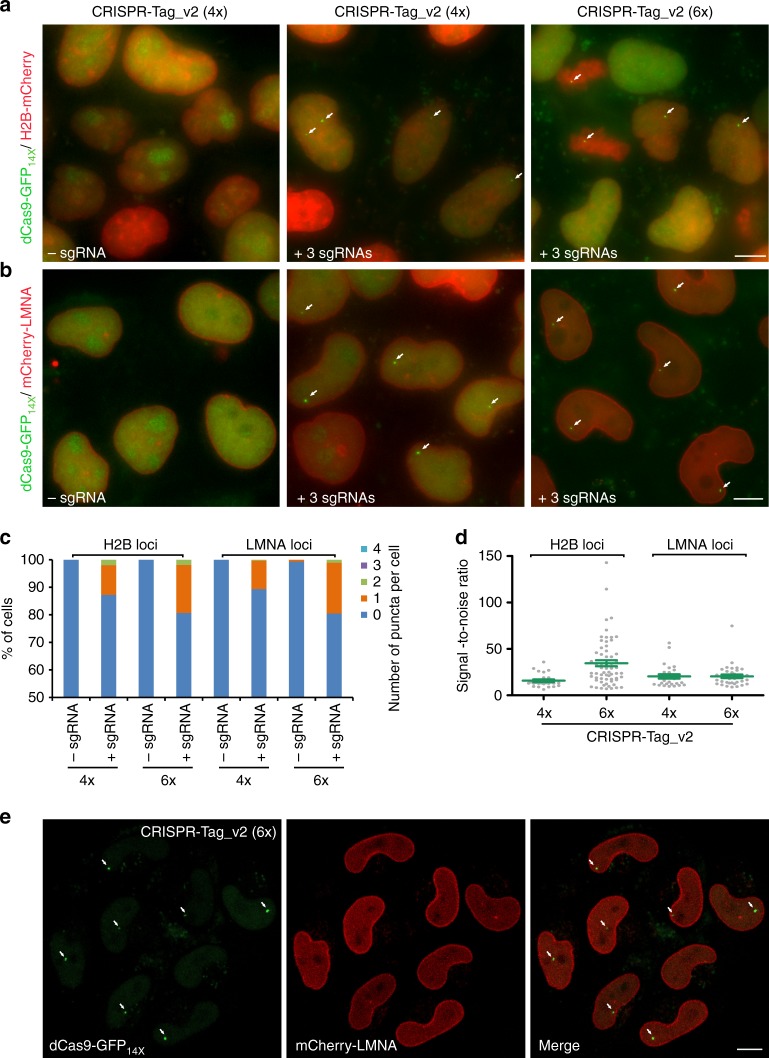
CRISPR-Tag in intron enables simultaneous visualization of DNA and protein of endogenous genes. **a** Visualization of H2B locus using CRISPR-Tag_v2 (containing four or six repeat units), which was inserted into the intron of mCherry. H2B-mCherry was imaged and H2B loci were labeled by dCas9-GFP_14X_ with three sgRNAs (sgTS5–sgTS7). **b** Visualization of LMNA locus using CRISPR-Tag_v2 with three sgRNAs (sgTS5–sgTS7). **c** Labeling efficiency of H2B and LMNA loci shown in **a** and **b**, respectively, was determined by counting the number of spots in each cell, *n* ≥ 191 cells. **d** Labeling efficiency of H2B and LMNA loci was determined by quantifying signal-to-noise ratio, *n* ≥ 25 cells. Green line denotes means ± SEM. **e** Confocal images of mCherry-LMNA and LMNA loci labeled by dCas9-GFP_14X_ and CRISPR-Tag_v2 (6x). All images in **a**, **b**, and **e** are maximum intensity projections from z stacks. Scale bars: 10 µm

There is no doubt that the longer the CRISPR-Tag is, the higher SNR can be achieved. Using CRISPR-Tag_v2_6x_ (six repeats, 635 bp) to label H2B locus, 19% of mix pool cells had clearly spots, while this number reduced to 10% using CRISPR-Tag_v2_4x_ (four repeats, 412 bp). Similar results were observed for labeling LMNA locus (Fig. 3a–c). Furthermore, CRISPR-Tag_v2_6x_ achieves relatively higher SNR, especially when labeling H2B locus (Fig. 3d). Obviously, the detection efficiency and quality can be further enhanced by using confocal microscopy (Fig. 3e). We performed short-term uninterrupted imaging to capture the dynamics of replicated LMNA loci and found that the CRISPR signal is stable (Supplementary Movie 2). Long-term live cell imaging was also carried out to track the dynamics of LMNA locus and mCherry-LMNA throughout the cell cycle (Supplementary Movie 3). Spinning-disk confocal imaging captured the entire process of breaking-down and reconstruction of mCherry-LMNA-labeled nuclear membrane, and also the duplication and separation of LMNA locus during mitosis. These results further demonstrate the applicability of our approach to capture long-term dynamics of DNA and protein of a specific gene throughout the cell cycle.

### Assess CRISPR-Tag designs for effective DNA labeling

**Fig. 4:**
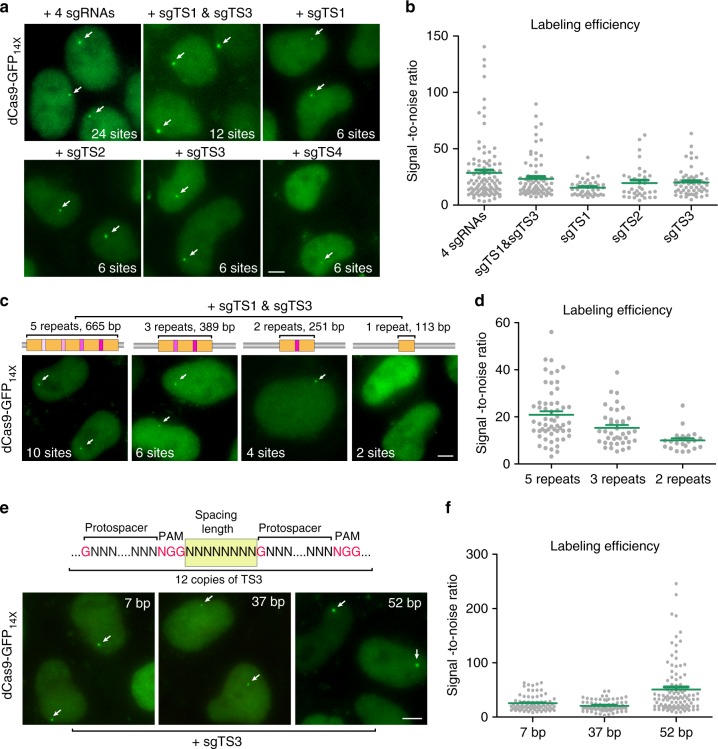
Assess CRISPR-Tag designs for effective DNA labeling. **a** Minimal number of sgRNAs to label H2B loci with CRISPR-Tag_v1. The use of four sgRNAs allows 24 binding sites of dCas9-GFP_14X_ and one sgRNA facilitates six CRISPR-targeting sites. Representative images were shown for each case. Detected H2B loci are indicated by arrows. **b** Comparison of labeling efficiencies of using different sgRNAs, *n* ≥ 33 cells. Error bars represent ±SEM. **c** Minimal number of CRISPR-targeting sites to label H2B loci. CRISPR-Tag containing different copies of repeat units were generated and then labeled by expressing both sgTS1 and sgTS3, respectively. **d** Labeling efficiency was defined by quantifying signal to noise ratio, *n* ≥ 23 cells. Green line reports means ± SEM. **e** Optimal spacing length between two neighboring targeting sites. Spacing length was defined as the number of nucleotides from NGG to next protospacer sequence as indicated in the diagram. **f** Labeling efficiency was defined by quantifying signal to noise ratio, *n* ≥ 72 cells. Green line reports means ± SEM. All images in **a**, **c**, and **e** are maximum intensity projections from z stacks. Scale bars: 5 µm

We next performed a series of optimizations and quantifications in order to reduce the size of the CRISPR-Tag while maintaining its labeling efficiency. First, we compared signal-to-noise ratio by using different numbers of sgRNAs to label CRISPR-Tag_v1, which is 803 bp long containing six repeats. Each repeat can be recognized by up to four sgRNAs (sgTS1–sgTS4). We observed that transfection of one single sgRNA, such as sgTS1, sgTS2, and sgTS3, resulted in sufficient visualization of CRISPR-Tag with SNR above 15. The SNR increased to 23.3 ± 8.7 and 28.6 ± 10.1 (mean ± SEM) when using two and four sgRNAs, respectively (Fig. 4a, b). Collectively, one sgRNA to target six sites is sufficient for labeling a specific locus. We next sought to further reduce the size of CRISPR-Tag by assembling fewer repeats, including 5, 3, 2, and 1 repeats. We found that the size of CRISPR-Tag can be as small as 251 bp harboring four CRISPR-targeting sites (Fig. 4c). However, it is important to note that shorter CRISPR-Tag resulted in lower SNR (Fig. 4d). Therefore, our results suggest using relatively longer CRISPR-Tag to achieve effective labeling. It is also important to optimize the spacing of two adjacent targeting sites in the CRISPR-Tag because steric hindrance of neighboring dCas9 protein-binding sites is a concern. We varied the spacing length and quantified SNR. The results indicated that 52 bp spacing linker separating the two neighboring targeting sites achieved the best result (Fig. 4e, f). Together, our studies provide some critical guidelines for CRISPR-Tag design. The smallest CRISPR-Tag is substantially shorter than the LacO/TetO array (usually ~10 and ~4 kb, respectively)^14^, and the ParB/INT (~ 1 kb) system^24^.

## Discussion

By assembling *C. elegans* genomic sequences which can be effectively targeted by the CRISPR-Cas9 system, we created CRISPR-Tag as a DNA tagging tool in living human cells, enabling live-cell labeling of non-repetitive genes at the level of single cells and single loci. Although these tags are as small as 251 bp, longer ones such as CRISPR-Tag_v2 (635 bp) do lead to higher SNR, which would be the optimal choice for most applications. Our CRISPR-Tags are consisted of no more than six repeats (24 CRISPR target sites). For comparison, a recent paper demonstrated an optimized TetO repeat for labeling endogenous loci while emphasizing that 96 repeats of TetO was required for long-term imaging^25^. The small size and low-repetitiveness of our CRISPR Tags are advantageous because they are less likely to perturb the chromatin structure or affect transcription and replication. It is possible that the repeat number of TetO can also be reduced using our signal amplification approach, although whether the 14x GFP labeling of TetR will affect its affinity to TetO needs to be tested.

All the CRISPR-Tags we created in this study are smaller than 850 bp and as small as 251 bp. Thus, CRISPR-Tag represents the smallest DNA tags among all the available tags. As a proof of concept, we developed a DNA tagging strategy, engineering CRISPR-Tag in the intron region of a fluorescent protein. Although this approach requires modification of endogenous loci, it has no significant effect on both gene transcription and protein expression, at least for the labeling of human H2B and LMNA. Systematic examination of even more protein-coding genes could further confirm this characteristic. Most importantly, our strategy enables simultaneously imaging of both DNA and protein expression of a specific gene. Thus, this imaging platform will provide a tool to uncover how gene positioning and movement play a role in gene regulation during development and disease progression.

An alternative ParB/INT system, also called “ANCHOR DNA labeling system (ANCHOR3)”, is based on insertion of a nonrepetitive DNA sequence (ANCH, ~1 kb) to which OR (bacterial partition protein or ParB) bind site-specifically^24^. Oligomerization of OR–FP fusion proteins lead to accumulation of fluorescent proteins, which non-specifically and dynamically associate with adjacent DNA. Although AHCHOR DNA tag is short and non-repetitive, whether the spreading of OR proteins to adjacent genomic region has any effects on the target region need to be carefully addressed. In addition, it is unknown whether AHCHOR DNA tag can work well for labeling endogenous genes in human cells. What remains to be done is the direct comparing of all the different DNA tags.

Our results indicated that sgRNAs successfully targeting *C. elegans* genome also work effectively in human cells. In theory, CRISPR-Tag approach can be applied broadly for any other species, such as using Human CRISPR-Tag for *C. elegans* genomic loci labeling. Furthermore, a same set of CRISPR-Tag and sgRNAs is applicable for tagging any genomic loci. This is an advantage over previous CRISPR imaging technique which requires at least 26 sgRNAs^6^. The labeling efficiency depends on how many of the sgRNAs are functional and how many are delivered into the cells. The number of sgRNAs required for non-repetitive DNA labeling might be reduced using super-resolution microscopy^9,10^. However, off-target or unspecific labeling needs to be carefully assessed if fewer sgRNAs are used. Combining dCas9-GFP_14X_ and CRISPR-Tag, DNA labeling was achieved by targeting 4–24 sites in our system. Obviously, the more target sites, the stronger signal would be detected.

Compared to previous CRISPR imaging technique, the disadvantage of CRISPR-Tag system is the additional knock-in step. Due to the low knock-in efficiency, we observed low frequency of clones with biallelic knock-in. This can be optimized by using novel CRISPR-mediated knock-in strategy, such as use of different types of donor DNA or different ways of repairing mechanisms^26,27^. Nevertheless, CRISPR-Tag provides DNA labeling at the level of single locus, which can be useful for some cases, such as labeling imprinted genes. One limit of CRISPR-Tag strategy is its use for tagging regulatory elements, such as enhancers, which is a general problem for all DNA tags. TetO repeats were integrated 16 kb far away from target genes^25^. ANCH tag was inserted adjacent to the promoter of the transgene^24^. To our knowledge, no DNA tags have been applied to label regulatory elements. Previous CRISPR imaging technique might be the best way to label regulatory elements. Important to note, an ideal DNA tagging system should not interfere with the function of the target loci. The binding of Cas9 to the target DNA begins with DNA unwinding to form an RNA–DNA hybrid, which may affect the localization of histones and other DNA-binding proteins^28,29^. However, Cas9 binding to DNA is reversible^30^, thus Cas9 could be replaced by other players. Our previous studies suggested dCas9 binding to telomeres did not apparently affect telomere integrity and their movement dynamics^6^. Nevertheless, the effects of dCas9 binding to the target loci remain to be fully addressed.

Orthologous CRISPR systems, with their different PAM requirements, provide a powerful platform for achieving multi-color labeling of genomic loci. Cas9 orthologs, SpCas9 and SaCa9, from *Streptococcus pyogenes* and *Staphylococcus aureus*, respectively, have been applied for labeling three different elements in one cell^31^. Alternatively, sgRNA scaffolds could be adapted to recruit different fluorescent proteins using MS2/PP7 motifs^32,33^. Thus, a wide range of orthogonal CRISPR-Tags with different CRISPR-targeting sequences can be assembled to achieve multi-color labeling. Considering that signal amplification using tandem split GFP greatly enhance the labeling efficiency, it would be useful to adapt SunTag^34^ system and split mCherry^35^ for generating more colors. Taken together, CRISPR-Tag opens up possibilities for both genomic tool developments and biological applications.

## Methods

### Cell culture

HeLa and HEK293T cells (obtained from UCSF Cell Culture Facility) were maintained in Dulbecco's modified Eagle medium (DMEM) with high glucose (Gibco) in 10% FBS (Clontech) and 1% penicillin/streptomycin (Gibco). All cells were grown at 37 °C and 5% CO_2_ in a humidified incubator.

### Construction of dCas9 and sgRNA

The nuclease-deficient *S. pyogenes* Cas9 (dCas9) was used for all the CRISPR imaging experiments in this study. The construction of dSpCas9-EGFP has been previously described^6^. To build dCas9-GFP11_14x_, we first synthesized two fragments of gBlocks, each containing seven copies of the coding sequence of GFP11 tag. Then we modified the SunTag vector (pHR-dSV40-NLS-dCas9-24xGCN4_v4-NLS-P2A-BFP-dWPRE, addgene #60910) to replace 24xGCN4 with GFP11_14x_. The DNA sequence encodes dCas9-GFP11_14X_ was provided in supplementary table 3. To express GFP1-10 in the nucleus, we fused GFP1-10 with one copy of SV40 NLS and cloned the fusion protein into a lentiviral vector containing a strong promoter *P*_SFFV._ The DNA sequence of GFP1-10 was provided in supplementary table 4. sgRNAs to label *MUC4* and 5S rDNA genes (Supplementary Figs. 2 and 3) were cloned into a lentiviral vector (AddGene #51024). Other sgRNAs in this study, including sgTS1–sgTS7, were built by modifying the CRISPRainbow vector (addgene #75398). For co-expression of multiple sgRNAs (>2), the Golden Gate Cloning method was used to assemble multiple sgRNAs into CRISPRainbow-donor vector (AddGene #75398)^32^. All the sgRNA-targeting sequences were listed in supplementary table 5.

### Assembly of CRISPR-Tag

To assemble the CRISPR-Tag, a gBlock containing a series of *C. elegans* genomic sequences (GNNNNNNNNNNNNNNNNNNNNGG) that can be recognized by CRISPR-Cas9 system was synthesized by Integrated DNA Technologies (IDT). The spacing of two neighboring CRISPR-targeting sits varies according to the design. To assemble a CRISPR-Tag with a desired number of repeats, the repeat unit containing 3 or 4 CRISPR-targeting sites was amplified by PCR reactions using the Phusion High-Fidelity DNA polymerase (New England Biolabs). The Golden Gate Cloning method was then performed to assemble CRISPR-Tags that consist of different desired numbers of repeats (diagram in supplementary Fig. 1). Important to note, there is a unique sequence arranged between two adjacent repeats in the CRISPR-Tag, which facilitates easy validation of CRISPR knock-in by PCR reactions. The sequences of CRISPR-Tag_v1 and CRISPR-Tag_v2 were provided in supplementary table 6.

### Re-engineering mCherry for carrying the CRISPR-Tag

The fourth intron of human HSPA5 gene was inserted into mCherry-coding sequence right after the three nucleotides that code for the 197th amino acid of mCherry protein. A restriction site BstXI was artificially embedded in the intron region for the ease of molecular cloning. CRISPR-Tag was then cloned into the BstXI site by In-Fusion HD Cloning Kit (Clontech). The sequences of mCherry fusions with intron and CRISPR-Tag were provided in supplementary table 7.

### Construction of donor plasmids

All donor plasmids used in this study were generated with NEBuilder HiFi DNA Assembly Cloning Kit (New England Biolabs). For example, to construct the donor plasmid for inserting mCherry and CRISPR tag to the C-terminus of H2B, the left and right homology arm (HA) were amplified from the genomic DNA of HeLa cells, with the stop codon being removed. Other two fragments, mCherry and CRISPR tag were amplified. Then the four fragments, including left HA, mCherry, CRISPR-Tag, and right HA were assembled into a same vector (diagram in Supplementary Fig. 1). To generate donor plasmids harboring sgH2B recognition sites, termed double-cut donor plasmid, the sgH2B-targeting sequence together with the PAM sequence (GCGAGCGCCAGGTCCCGGCAGGG) was included in the forward primer of left HA and the reverse primer of right HA. Therefore, sgH2B-targeting sequence was tagged to the regions flanking the upstream and downstream HA.

### Lentiviral production

HEK293T cells were seeded into six-well plates 24 h prior to transfection. 110 ng of pMD2.G plasmid, 890 ng of pCMV-dR8.91, and 1000 ng of the lentiviral vector (dCas9-EGFP, dCas9-GFP11_14x_, GFP1-10 or sgRNA) were co-transfected into HEK293T in each well using FuGENE (Promega) following the manufacture's recommended protocol. Virus was harvested 48 h after transfection.

### Transfection, infection, and generation of stable cell lines

HeLa cells were infected with dCas9-EGFP and Tet-on 3G lentiviruses, and then clonal cell lines were isolated to express dCas9-EGFP at a suitable level following our previous protocol^36^. We diluted the cells and distributed 0–1 cell into each well of 96-well plate. After 10–14 days, selected colonies were tested for DNA labeling. The basal expression level of dCas9-EGFP without doxycycline induction is ideal to achieve high signal-to-noise ratio. To generate HeLa cells stably expressing dCas9-GFP_14x_, we infected HeLa cells with dCas9-GFP_14x_ and GFP1-10 lentivirues. A clonal cell line which achieved the best signal-to-noise ratio was selected for CRISPR imaging. The non-repetitive region of *MUC4* gene was labeled by infecting dCas9-GFP expressing cells with a mixed lentiviral cocktail containing 36 sgRNAs. 3 μg of the purified plasmid cocktail (5–6 sgRNAs per cocktail), 0.33 μg of pMD2.G and 2.66 μg of pCMV-dR8.91 were cotransfected into HEK293T cells seeded in T25 flask for creating a mixed lentiviral cocktail. 48-h postransfection, seven lentiviral cocktails for these 36 sgRNAs were generated and mixed (with equal amounts of each cocktail). dCas9-GFP-expressing cells were then infected with lentiviral mixtures in eight-well of chambered coverglass. To enhance the infection of each lentivirus, a higher dosage of virus (2:3 dilution) in the presence of polybrene (5 μg/mL) was used for non-repetitive DNA labeling. To label repetitive genomic elements or specific protein-coding genes, dCas9-FP stable cell line without/with successful knock-in of CRISPR-Tag was transfected with sgRNAs using FuGENE (Promega) following the manufacturer's recommended protocol. For all the CRISPR imaging experiments, HeLa cells were plated into eight-well chambered coverglass (Lab-Tek II). 400 ng of total sgRNA plasmid DNA were transfected for each individual well.

### sgRNA in vitro transcription

sgRNAs for CRISPR-mediated knock-in was transcribed in vitro following the published protocol^37^. The following sequence was used as the DNA template to transcribe sgRNAs in vitro: 5′-TAA TAC GAC TCA CTA TAG GNN NNN NNN NNN NNN NNN NNG TTTAAG AGC TAT GCT GGA AAC AGC ATA GCA AGT TTA AAT AAG GCT AGT CCG TTA TCA ACT TGA AAA AGT GGC ACC GAG TCG GTG CTT TTT TT-3′ containing a T7 promoter (TAATACGACTCACTATAG), a gene specific ∼20-nt protospacer sequence starting with a G for optimal T7 transcription (GNNNNNNNNNNNNNNNNNNN), and a common sgRNA scaffold region. The DNA template was generated by overlapping PCR using a set of four primers: three primers common to all reactions (forward primer T25: 5′-TAA TAC GAC TCA CTA TAG-3′; reverse primer BS7: 5′-AAA AAA AGC ACC GAC TCG GTG C-3′ and reverse primer ML611: 5′-AAA AAA AGC ACC GAC TCG GTG CCA CTT TTT CAA GTT GAT AAC GGA CTA GCC TTA TTT AAA CTT GCT ATG CTG TTT CCA GCA TAG CTC TTA AAC-3′) and one gene-specific primer (forward primer 5′-TAA TAC GAC TCA CTA TAG GNN NNN NNN NNN NNN NNN NNG TTT AAG AGC TAT GCT GGA A-3′). For each template, a 100 μL PCR was set using iProof High-Fidelity Master Mix (Bio-Rad) reagents. The PCR product was then purified using DNA Clean and Concentrator-5 columns (Zymo primer (forward primer 5′-TAA TAC GAC TCA CTA TAG GNN NNN NNN NNN NNN NNN NNG TTT AAG AGC TAT GCT GGA A-3′). For each template, a 100 μL PCR was set using iProof High-Fidelity Master Mix (Bio-Rad) reagents. The PCR product was then purified using DNA Clean and Concentrator-5 columns (Zymo Research) following the manufacturer’s instructions. Next, a 100 μL in vitro transcription reaction was carried out using HiScribe T7 High Yield RNA Synthesis Kit (New England Biolabs). The sgRNA product was then purified on RNA Clean and Concentrator-5 columns (Zymo Research) and eluted in 15 μL of RNase-free RNA buffer (10 mM Tris pH 7.0 in DEPC-treated H_2_O). sgRNA quality was examined by running 3 pg of the purified sgRNA on a 10% polyacrylamide gel containing 7 M urea (Novex TBE-urea gels, ThermoFisher Scientific).

### CRISPR-mediated knock-in

RNP assembly and electroporation were performed to carry out CRISPR knock-in experiments. Cas9/sgRNA RNP complexes were prepared following methods reported by Lin et al.^21^. Cas9 protein (pMJ915 construct, containing two nuclear localization sequences) was expressed in *E. coli* and purified by the University of California Berkeley Macrolab following protocols described by Jinek et al.^3^. The HeLa dCas9-GFP_14x_ cells were treated with 200 ng/mL nocodazole (Sigma) for 15 h prior to electroporation to increase HDR efficiency. RNP complexes were assembled with 100 pmol Cas9 protein and 130 pmol sgRNA just before electroporation and combined with 400 ng donor plasmid DNA in a final volume of 10 μL. First, 130 pmol-purified sgRNA was diluted in Cas9 buffer (final concentrations: 150 mM KCl, 20 mM Tris pH 7.5, 1 mM TCEP–HCl, 1 mM MgCl_2_, 10% vol/vol glycerol) and incubated at 70 °C for 5 min. A total of 2.5 μL of Cas9 protein (40 μM stock in Cas9 buffer, i.e., 100 pmol) was then added and RNP assembly was carried out at 37 °C for 10 min. Finally, donor plasmid DNA was added to this RNP solution. Electroporation was performed in a Amaxa 96-well shuttle Nuleofector device (Lonza) using SF-cell line reagents (Lonza) following the manufacturer’s instructions. Nocodazole-treated HeLa cells were washed with PBS and resuspended to 10^4^ cells per microliter in SF solution immediately before electroporation. For each sample, 20 μL of cells (i.e., 2 × 10^5^ cells) was added to the 10 μL RNP/template mixture. Cells were immediately electroporated using the CM130 program and transferred to 1 mL pre-warmed culture medium in a 24-well plate. FACS selection of knock-in positive cells were carried out 3 days after electroporation.

### Flow cytometry

Three days following Cas9/sgRNA/donor electroporation, cells were analyzed by flow cytometry on aLSR II instrument (BD Biosciences) and sorted on a FACSAria II (BD Biosciences). Cells were first gated for the intact cell population using forward scatter versus side scatter plots and then gated for single cells based on forward scatter W versus forward scatter H. mCherry-positive cells were sorted out for further validation of CRISPR knock-in.

### Quantitative RT-PCR

Cells were harvested using trypsin (Hyclone), and total RNA was isolated using FastPure Cell/Tissue Total RNA Isolation Kit (Vazyme), according to manufacturer’s instructions. RNA was converted to cDNA using oligo-dT primers (HiScript II Q RT SuperMix for qPCR, Vazyme). Quantitative PCR reactions were prepared using ChamQ Universal SYBR qPCR Master mix (Vazyme). Reactions were run on the QuantStudio 5 Real-Time PCR system (Thermo Fisher). All reactions were performed in triplicate. RNA abundance was normalized to an endogenous reference gene UBC and calculated as delta–delta threshold cycle (ΔΔCt). Primers used for q-RT-PCR are listed in Supplementary Table 8.

### Microscopy and data analysis

CRISPR imaging data were acquired on a Nikon Ti-E inverted wide-field fluorescence microscope equipped with a ×100 NA 1.40 PlanApo oil immersion objective, an LED light source (Excelitas X-Cite XLED1), an sCMOS camera (Hamamatsu Flash 4.0), and a motorized stage (ASI) with stage incubator (Tokai Hit). Acquisitions were controlled by MicroManager. All images were taken as z stacks at 0.4 μm steps and with a total of 15 steps and were projected in maximum intensity. Long-term live cell imaging was performed on Andor Dragonfly (high-speed confocal microscopy) based on Nikon TI microscope with Nikon Perfect Focus system, ×60 NA 1.40 objective, an Andor iXon Ultra 888 EM-CCD camera. Images were taken as z stacks at 0.5 μm steps (7 steps) and at a frame rate of 5 Hz. Interval time was set as 10 min. Cells were imaged for 4–6 h. During image acquisition, cells ware maintained at constant temperature of 37 °C and 5% CO_2_ within an incubation box. All the fluorescence imaging data were analyzed by Image J. Signal-to-noise ratio was defined as the ratio of the intensity of a fluorescent signal and the power of background noise as following formula:

$${{\rm{SNR}}}={\frac{P_{{{{\rm{signal}}}}}}{P_{{{{\rm{noise}}}}}}}={\frac{{\rm{Max}}\,{\rm{intensity}}\,{\rm{of}}\,{\rm{GFP}\,{\rm{spot}}}-{\rm{Mean}}\,{\rm{intensity}}\,{\rm{of}}\,{\rm{background}}\,{\rm{GFP}}}{{\rm{Std.}}\,{\rm{dev.}}\,{\rm{of}}\,{\rm{background}}\,{\rm{signal}}}}$$

## Electronic supplementary material

Supplementary Information

Supplementary Movie 1

Supplementary Movie 2

Supplementary Movie 3

Description of Additional Supplementary Files

## Data Availability

The authors declare that all the data supporting the findings of this study are available from the authors on reasonable request.
