# Supplementary material for: Efficient labeling and imaging of protein-coding genes in living cells using CRISPR-Tag
Source: Nat Commun. 2018 Nov 29;9:5065. doi: 10.1038/s41467-018-07498-y (PMC6265289; doi:10.1038/s41467-018-07498-y)
Supplement: Supplementary file 1 — Supplementary Information [file 41467_2018_7498_MOESM1_ESM.pdf]

Efficient Labeling and Imaging of Protein-coding Genes in Living Cells using CRISPR-Tag

Chen et al.

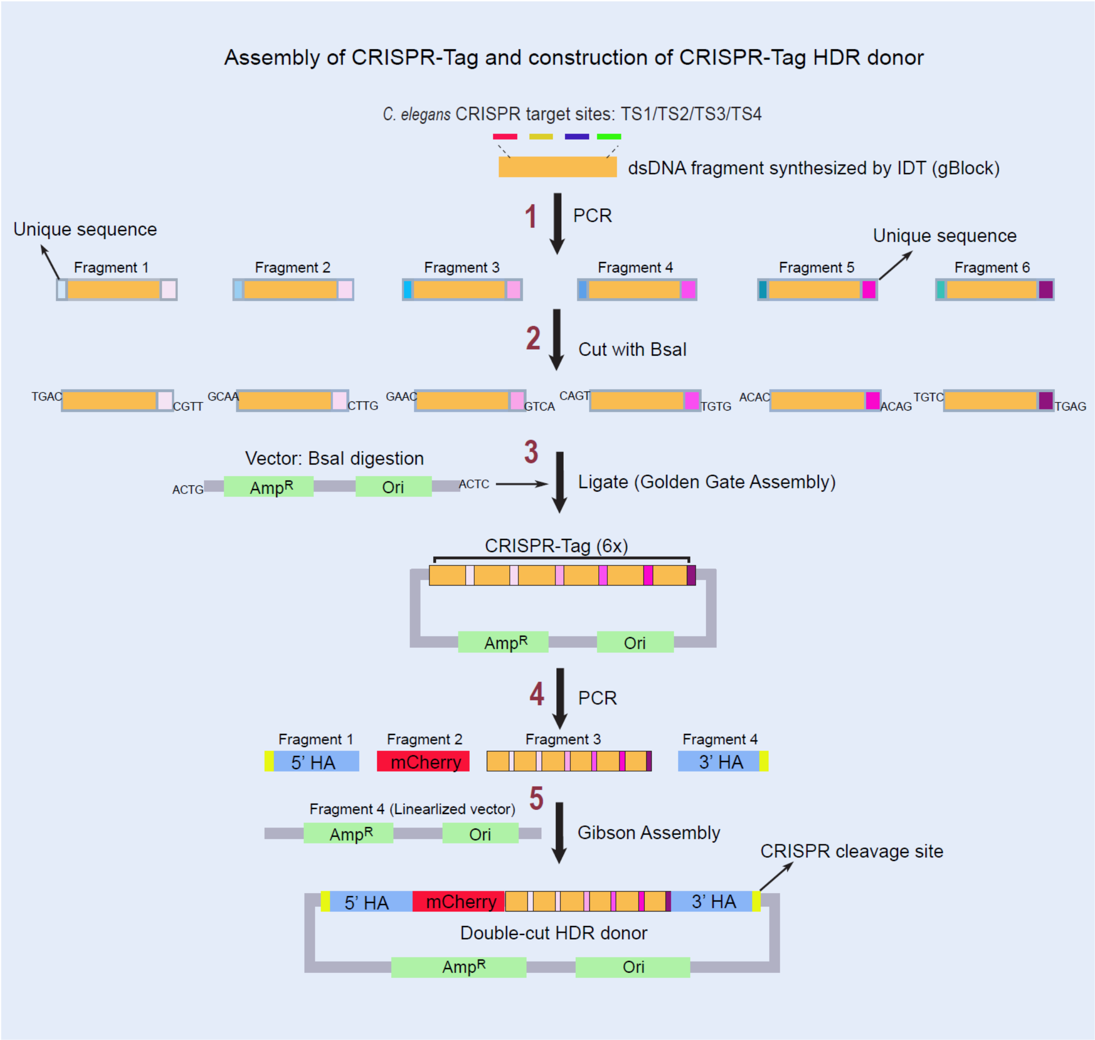

Supplementary Figure 1

Assembly of CRISPR-Tag and HDR donor (related to Fig. 1).

A gBlock containing four *C. elegans* genomic sequences (TS1/TS2/TS3/TS4) that can be recognized by CRISPR-Cas9 system was synthesized by Integrated DNA Technologies (IDT). Assembly of CRISPR-Tag involves three steps. Setp 1: The repeat unit of CRISPR-Tag was amplified by PCR reactions using six pairs of primers, respectively. Each PCR fragment contains unique sequences at both 5' and 3' end, harboring BsaI sites. Step 2: PCR fragments and sgRNA vector were digested by BsaI. Step 3: The Golden Gate Cloning method was performed to assemble the CRISPR-Tag. At the end, there is a unique sequence arranged between every two adjacent repeats in the CRISPR-Tag, which facilitates easy validation of CRISPR knock-in by PCR reactions. Step 2 and Step 3 can be completed in a same tube. Construction of CRISPR-Tag HDR donor involves two steps. First, 5'/3' homology arms (HA), mCherry and CRISPR-Tag were amplified by PCR reactions. Second, all the inserts and the vector were assembled by Gibson Assembly method.

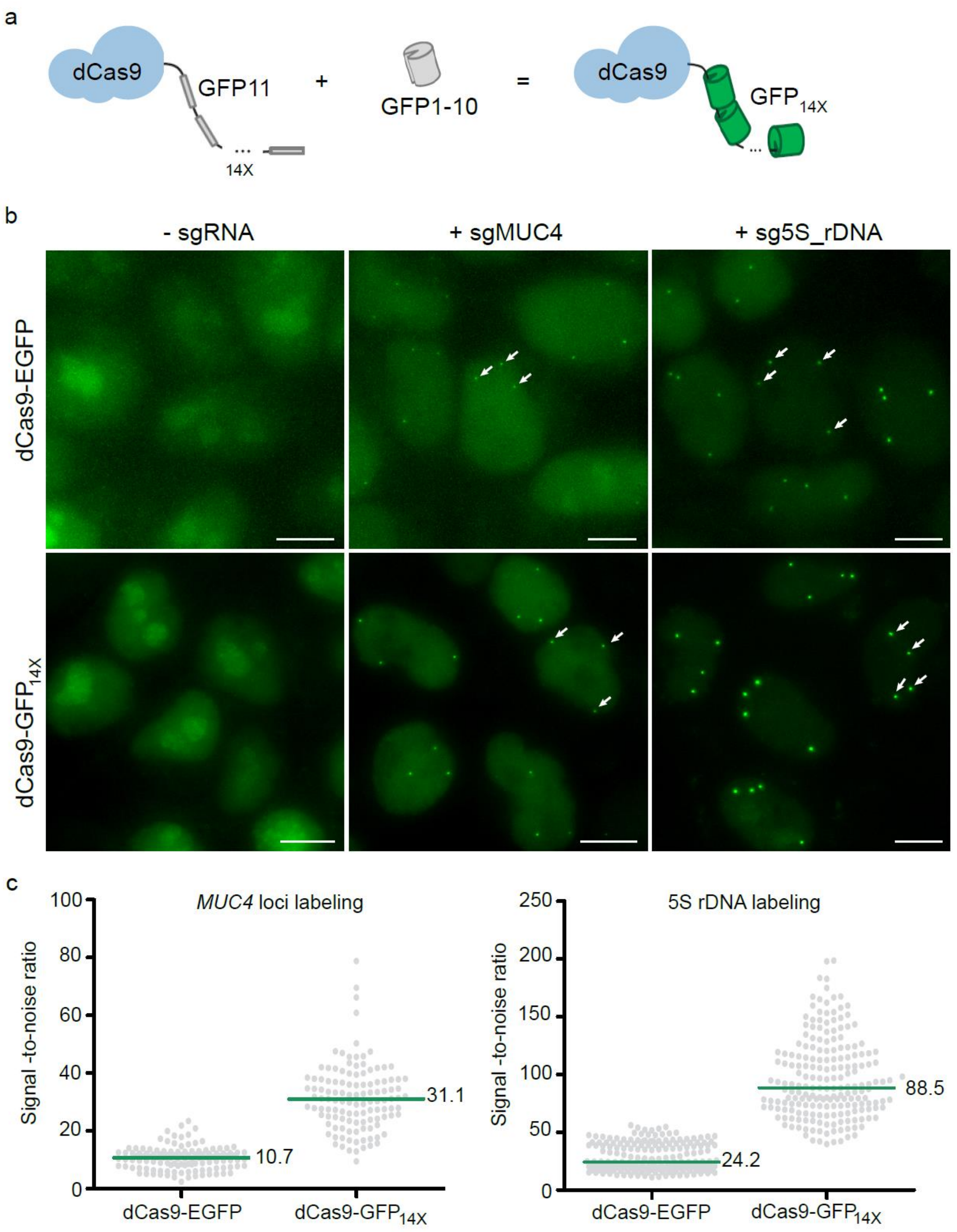

Supplementary Figure 2

dCas9-GFP$_{14X}$ allows efficient labeling of genomic loci (related to Fig. 1).

(a) Schematic of fluorescence signal amplification by tandem GFP11. (b) Representative images demonstrate *MUC4* and 5S rDNA labeling using dCas9-EGFP or dCas9-GFP$_{14X}$. All images are maximum intensity projections from z stacks. Scale bars: 10 µm. (c) Quantifications of signal-to-noise ratio to compare labeling efficacies. Each dot represents a single cell. Green line indicates the median value, n ≥ 119 cells.

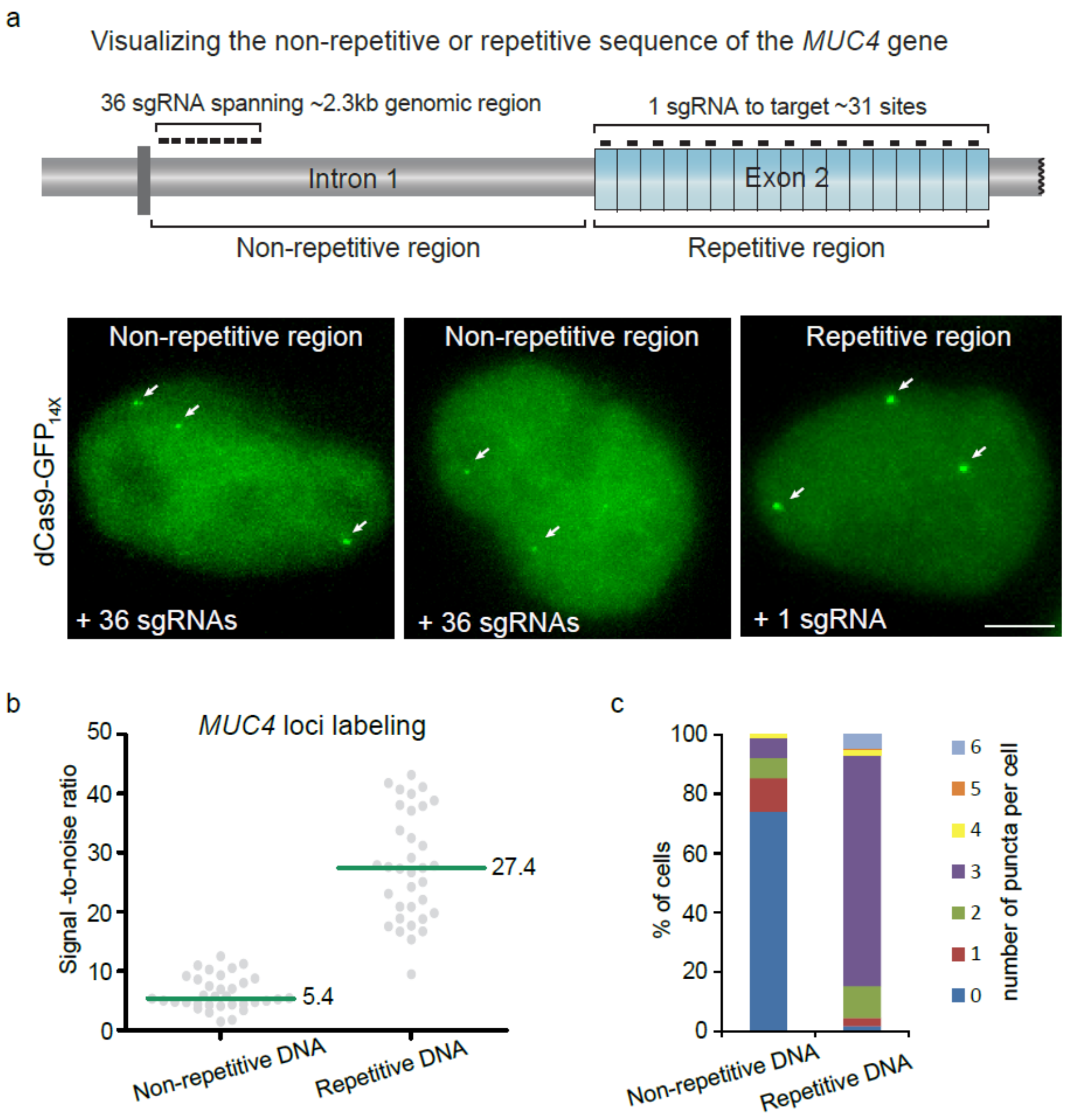

Supplementary Figure 3

Labeling *MUC4* gene in the non-repetitive or repetitive DNA region (related to Fig. 1).

(a) Labeling of *MUC4* intron 1 and exon 1 by CRISPR imaging. Non-repetitive intron 1 was labeled by using 36 sgRNAs, whereas repetitive exon 1 was labeled by using only one sgRNA. 2 to 3 spots (arrows) can be detected. All images are maximum intensity projections from z stacks. Scale bars: 5 µm. (b) Labeling efficiency of non-repetitive and repetitive region in *MUC4* gene was compared by measuring the signal-to-noise ratio. Dots represent individual cells. Green line denotes the median value, n = 34 cells. (c) Labeling efficiency of *MUC4* loci was determined by counting the number of spots in each cell. Non-repetitive DNA, n = 265; Repetitive DNA, n = 289.

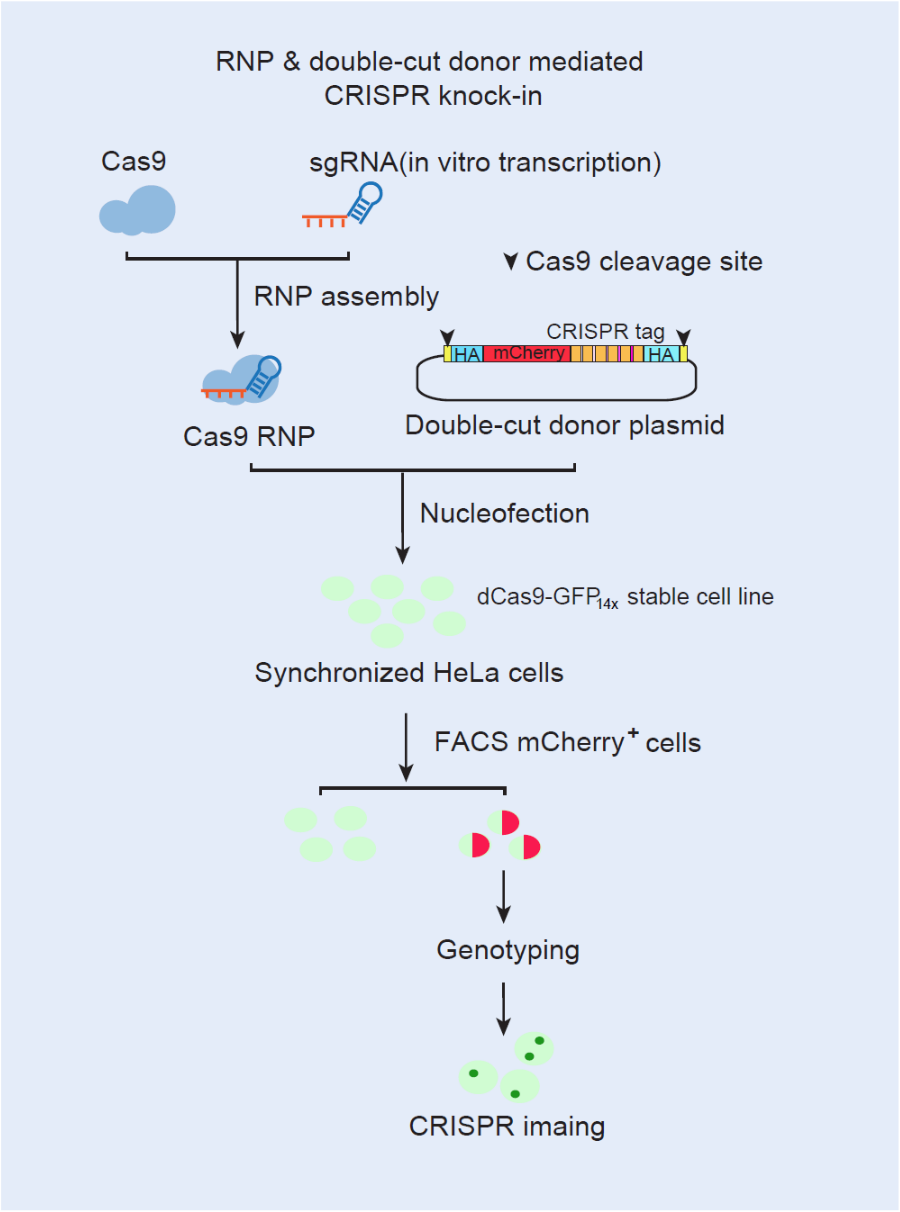

Supplementary Figure 4

Experimental work flow of CRISPR-Tag based DNA tagging (Related to Fig. 1, 2 and 3).

First, CRISPR-Tag together with mCherry was inserted into a specific locus by CRISPR-Cas9 mediated knock-in. The knock-in experiments were carried out by electroporation of Cas9/sgRNA ribonucleoprotein (RNP)/HDR donor into HeLa cells. Left homology arm, CRISPR-Tag, mCherry and right homology arm were assembled as a donor plasmid harboring two Cas9 cleavage sites as indicated. Four days after nucleofection, mCherry^{+} cells, which represent knock-in^{+} cells were sorted out by FACS. Genotyping was then performed to confirm the successful knock-in. The final step was to transfect sgRNAs into the cells for CRISPR labeling.

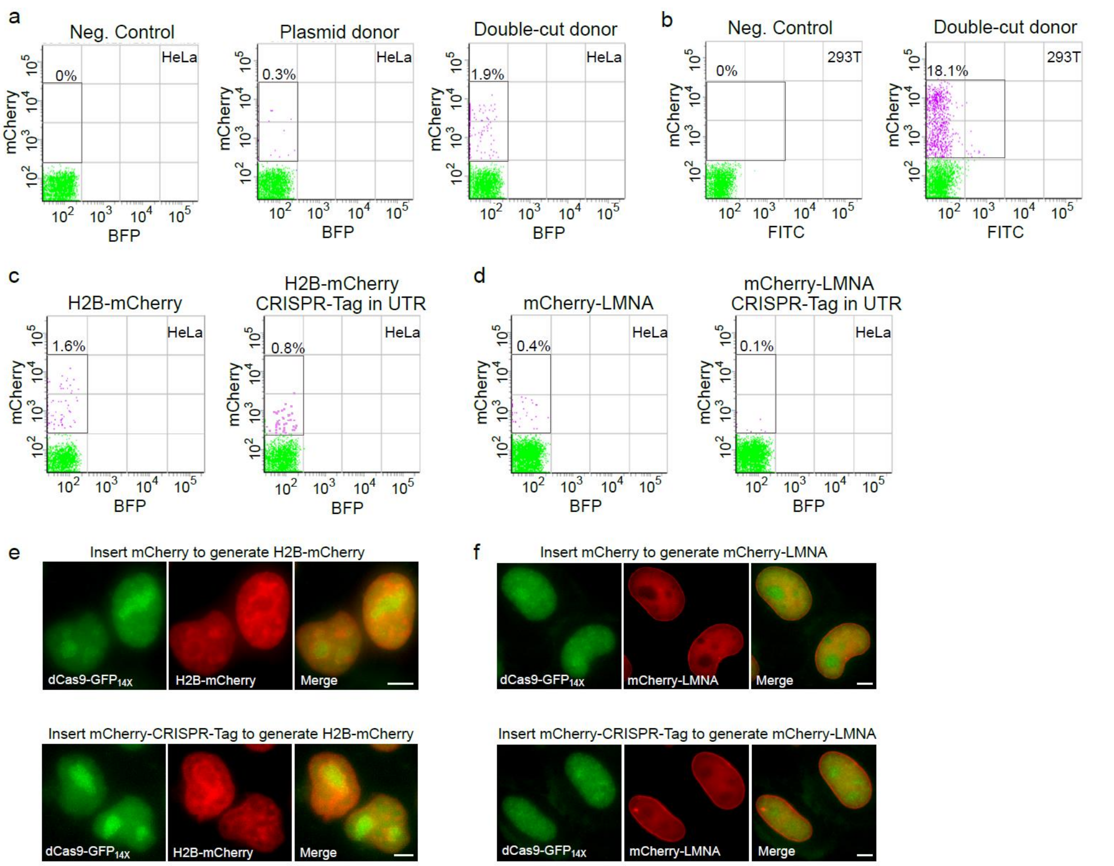

Supplementary Figure 5

Quantitative analysis of HDR efficiency (Related to Fig. 1).

(a-d) FACS analysis was carried out four days after RNP/donor electroporation. BFP serves as an irrelevant channel. The percentage of mCherry+ cells represents the HDR-mediated knock-in efficiency. (a) mCherry was integrated to C-terminus of H2B locus by using conventional plasmid donor or double-cut plasmid donor in HeLa cells. Cells were first gated for the intact cells by FSC/SSC plot and then gated for single cells based on FSC-A/FSC-W. mCherry positive cells were established by first analyzing cells negative for mCherry and setting the gate for mCherry positive cells. (b) mCherry was inserted to C-terminus of H2B locus by using double-cut plasmid donor in 293T cells. (c) Left: mCherry was integrated to the C-terminus of H2B locus; Right: mCherry plus CRISPR-Tag were inserted to the C-terminus of H2B locus. (d) Left: mCherry was integrated to the N-terminus of LMNA locus; Right: CRISPR-Tag plus mCherry were inserted to the N-terminus of H2B locus. (e-f) $mCherry^{+}$ cells isolated by FACS were imaged to examine the subcellular localization of H2B-mCherry (e) and mCherry-LMNA (f). All images are maximum intensity projections from z stacks. Scale bars: 5 µm.

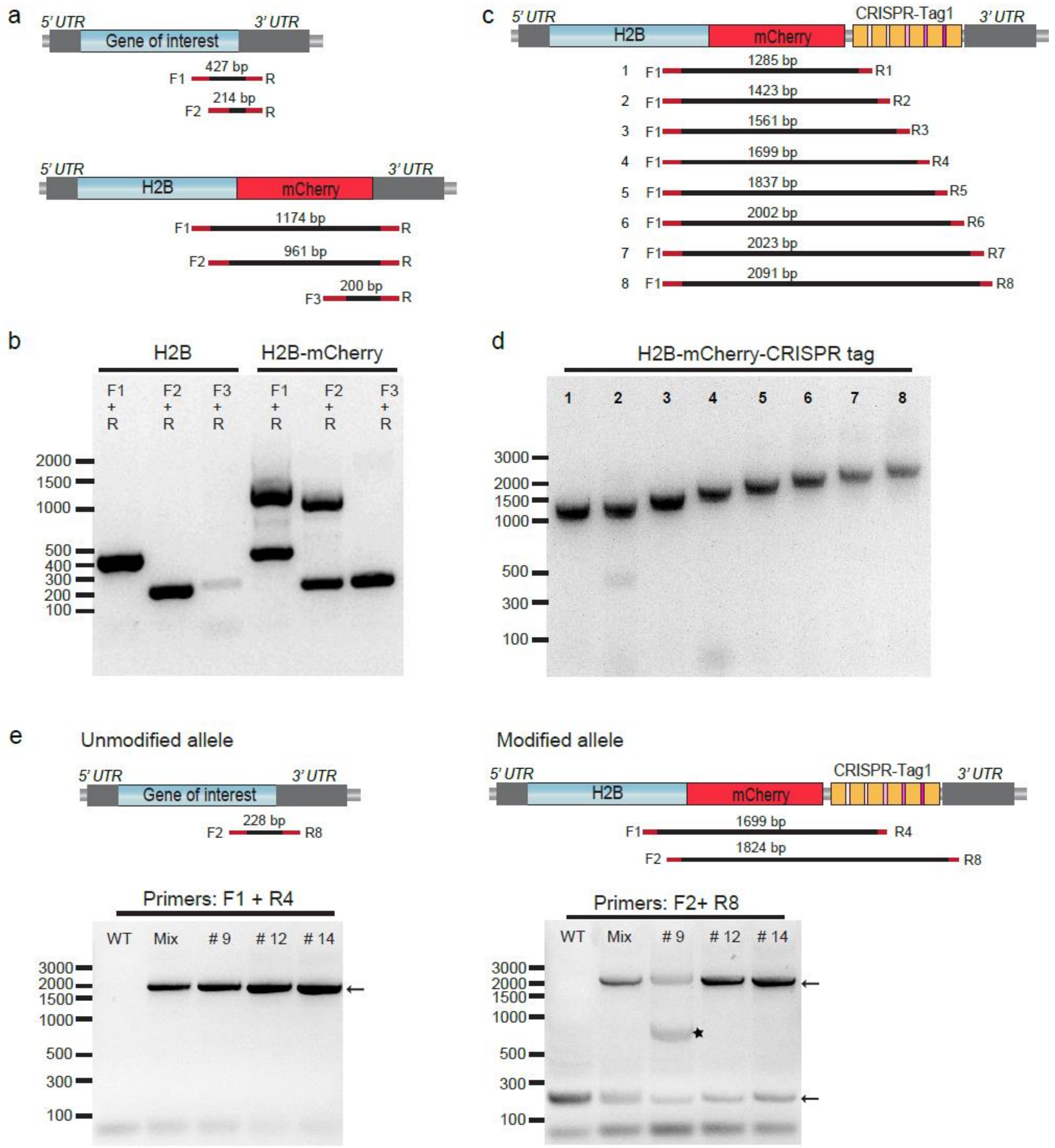

Supplementary Figure 6

PCR-based validation of CRISPR knock-in (Related to Fig. 1).

(a, c) Schematic of primer designs to validate mCherry (a) or mCherry-CRISPR-Tag (c) insertion at the C-terminus of H2B. The size of each PCR fragment is indicated for every pair of primers. (b, d) Genomic DNA from each sample was purified and used as the PCR template. Representative gels of PCR products are shown to indicate the correct insertions. The size of all PCR products is correct as expected. (e) Genomic DNA PCR was performed for Clone 9, 12 and 14 by using indicated primers. Arrows indicated the expected PCR products and the star highlighted an extra band in Clone 9. Sequencing results suggested that Clone 9 may contain two CRISPR-Tag modified alleles. One allele harbors a full sequence of mCherry-CRISPR-Tag, while another allele only contains part of CRISPR-Tag sequence, which might be resulted in genomic rearrangement in HeLa cells.

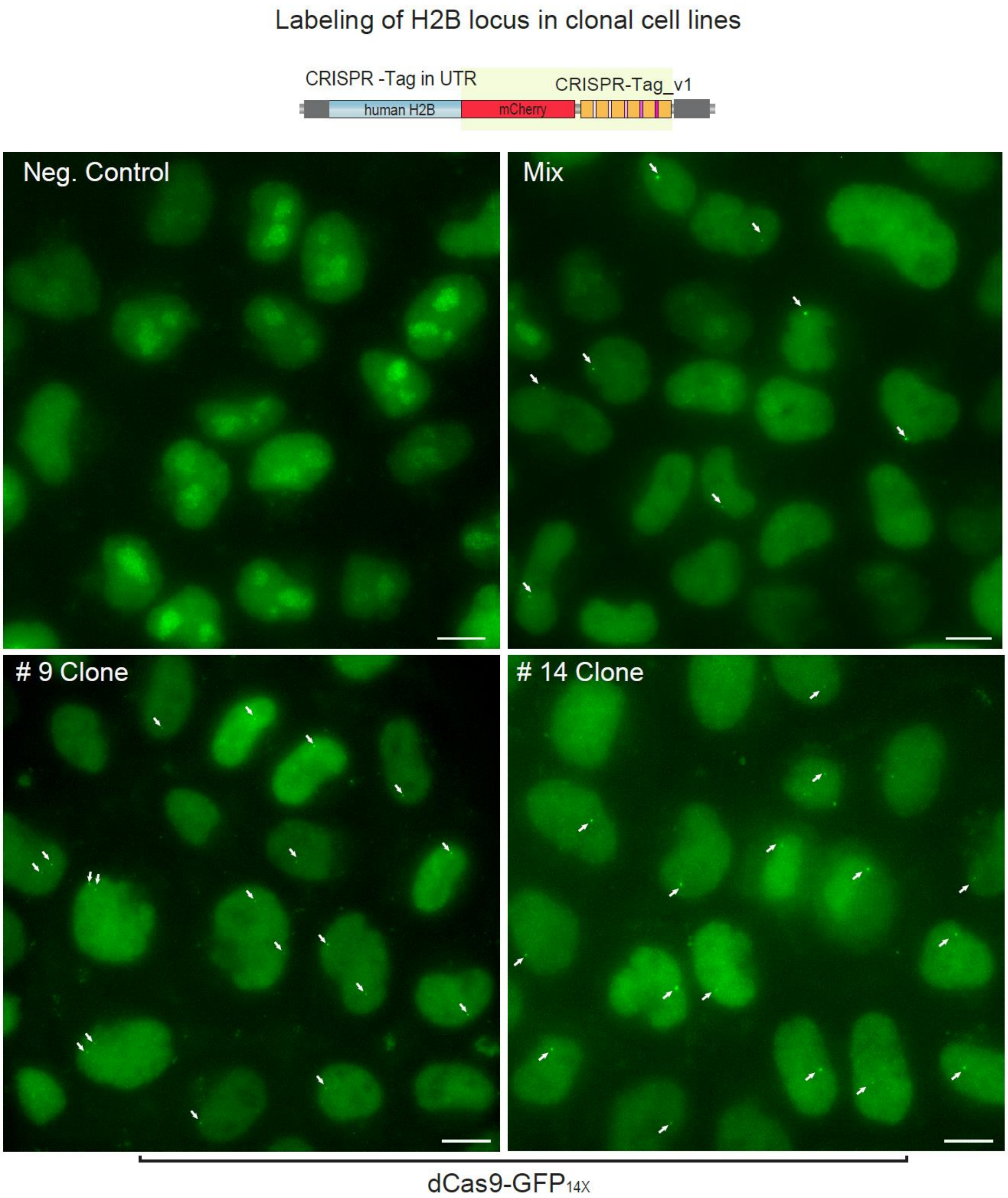

Supplementary Figure 7

Labeling of H2B loci in mix pool cells and clonal cell lines (Related to Fig. 1).

Large field of images to show the labeling efficiency of H2B loci in mix pool and clonal cells, including Clone 9 and Clone 14. The sgRNA vector co-expressing four sgRNAs was transfected to label H2B loci. Images were acquired on wide-field fluorescent microscopy using 100x NA 1.40 PlanApo oil immersion objective. Arrows indicate the spots which represent H2B loci. All images are maximum intensity projections from z stacks. Scale bars: 10 µm.

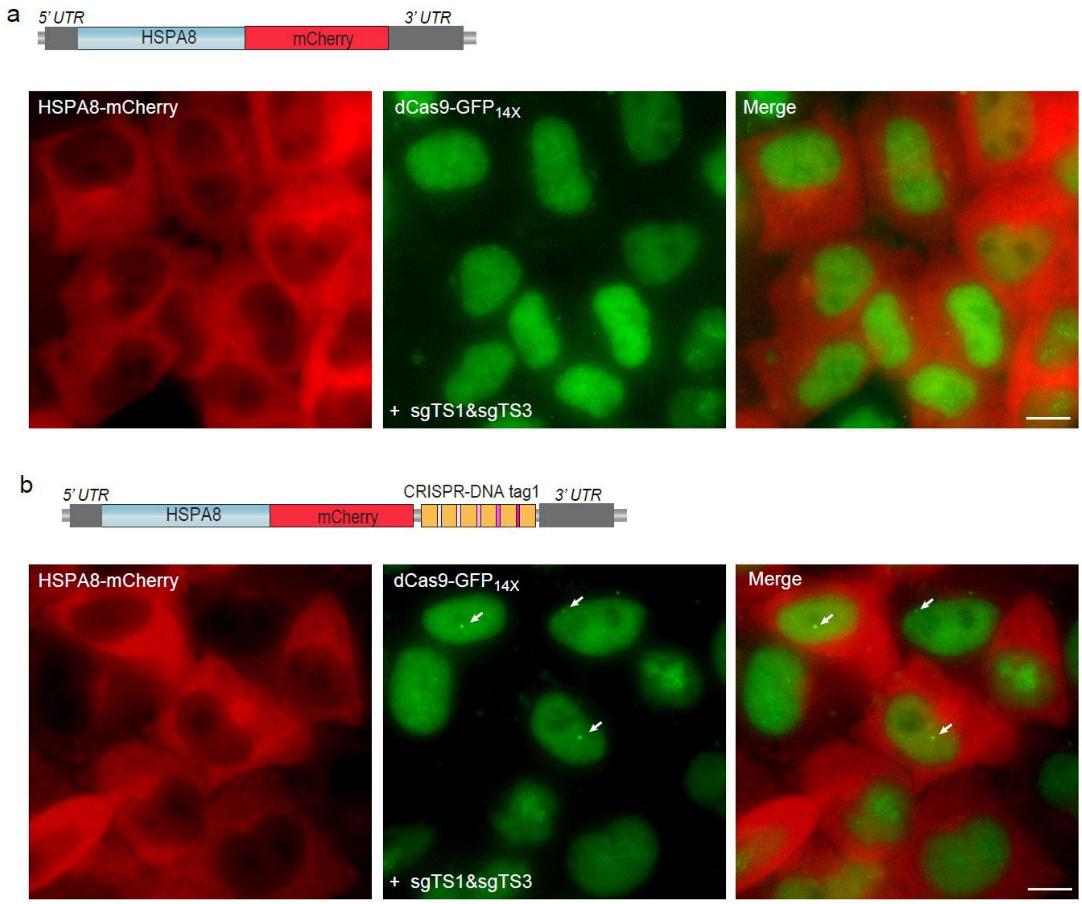

Supplementary Figure 8

Application of CRISPR-Tag to label human HSPA8 gene (Related to Fig. 1).

(a) Human HSPA8 gene codes a member of the heat shock protein 70 family. mCherry was integrated to the C-terminus of HSPA8. HSPA8-mCherry is mainly localized to the cytoplasm as shown. Transfection of sgRNAs did not result in specific fluorescent spots. (b) mCherry-CRISPR-Tag was inserted at the C-terminus of HSPA8. HSPA8 loci could be observed by co-expressing sgTS1 and sgTS3. All images are maximum intensity projections from z stacks. Scale bars: 10 µm.

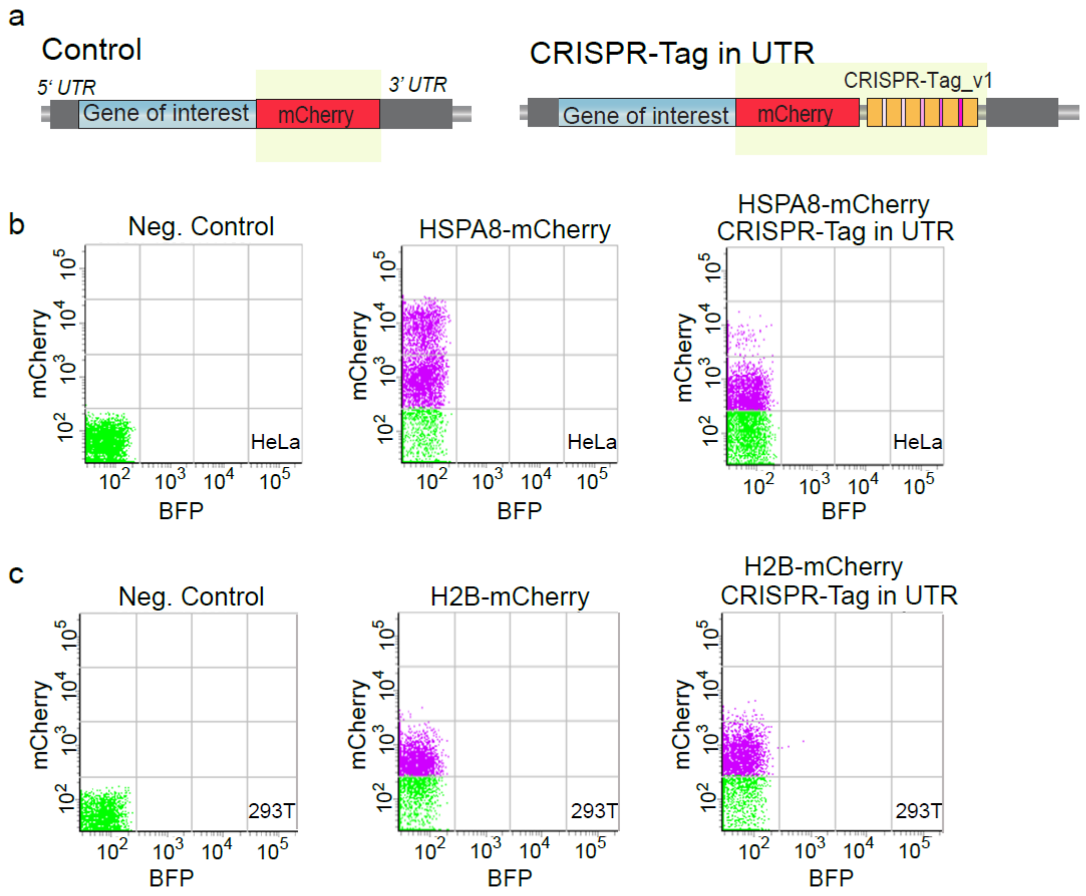

Supplementary Figure 9

Insertion of CRISPR-Tag in the UTR region down regulates protein expression in HeLa cells (Related to Fig. 2).

(a) Schematic of mCherry or CRISPR-Tag_v1 insertion to a genomic locus of interest. The CRISPR knock-in fragments are highlighted in yellow. (b) Quantitative analysis of FACS indicates mCherry intensity of individual HeLa cells for three conditions, control, mCherry and CRISPR-Tag-mCherry integration at the C-terminus of HSPA8. (c) Quantitative analysis of FACS to report H2B-mCherry expression in 293T cells for three conditions, control, mCherry and mCherry-CRISPR-Tag integration at the C-terminus of H2B. For all FACS plots, dots denote single cells. BFP serves as an irrelevant channel.

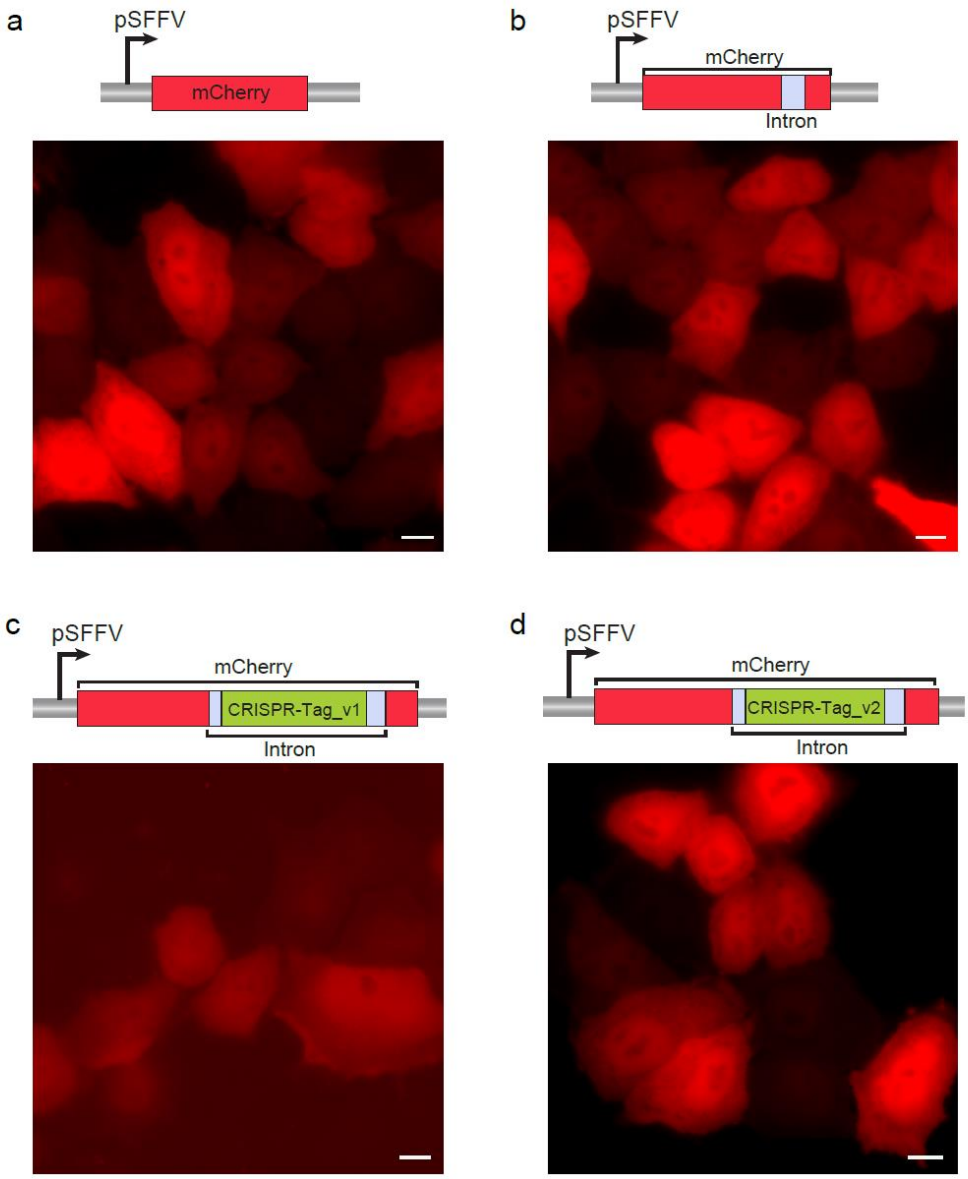

Supplementary Figure 10
Re-engineering mCherry-coding sequence with introns (Related to Fig. 2).

(a-d) Representative images of mCherry expression driven by a strong promoter at four different conditions: mCherry without introns (a), mCherry with one artificial intron (b), mCherry with one intron harboring CRISPR-Tag_v1 (c) and mCherry with one intron carrying CRISPR-Tag_v2 (d). Overall expression of mCherry was dramatically decreased when CRISPR-Tag_v1 was included in the intron region, whereas CRISPR-Tag_v2 included in the intron did not affect mCherry expression. All images in are from a single focal plane. Scale bars: 10 µm.

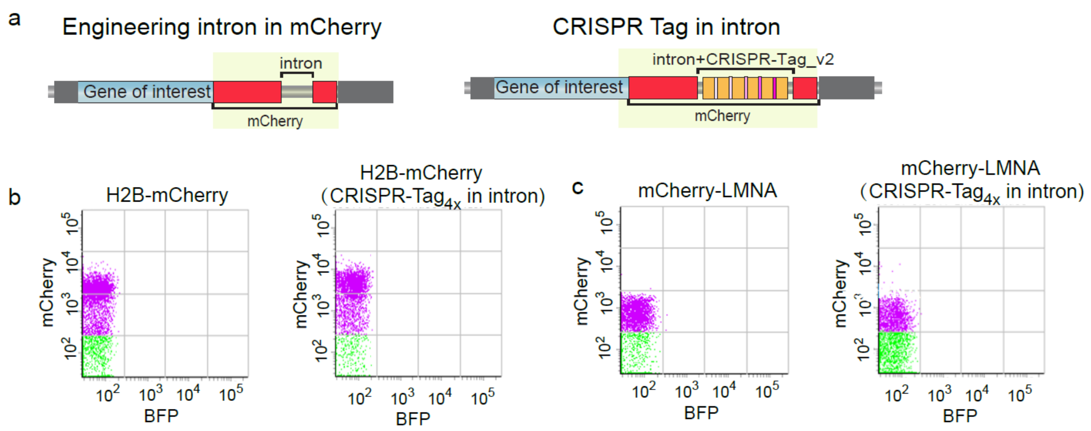

Supplementary Figure 11

Protein expression remains normal when CRISPR-Tag_v2 is embedded in the intron region (Related to Fig. 2).

(a) Schematic of mCherry or CRISPR-Tag_v2 insertion to a genomic locus of interest. (b) FACS quantitative analysis reports mCherry intensity of individual HeLa cells for the two conditions, integrations of mCherry, mCherry with intron plus CRISPR-Tag_v2 containing 4 repeats to the C-terminus of H2B, respectively. (c) Quantitative analysis of FACS was performed for the following conditions, integrations of mCherry, mCherry with intron plus CRISPR-Tag_v2 containing containing 4 repeats to the N-terminus of LMNA, respectively. Dots denote single cells. BFP serves as an irrelevant channel.

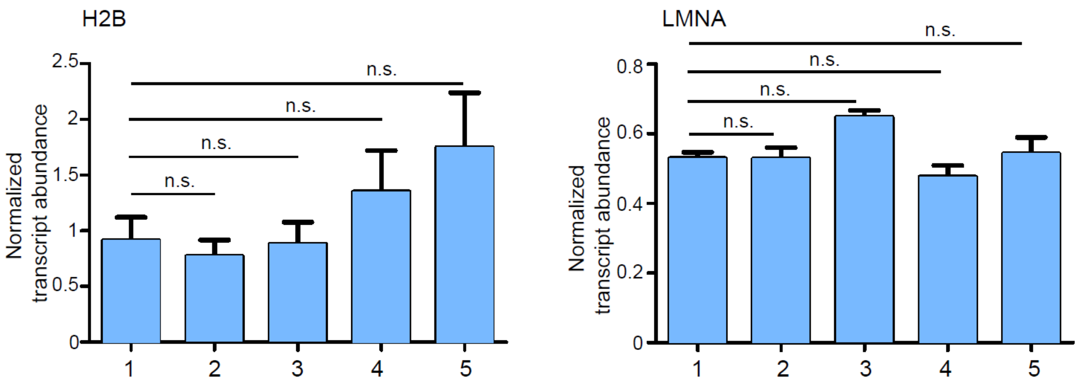

1: H2B-mCherry
2: H2B-mCherry-CRISPR-Tag_v1 in UTR
3: H2B-mCherry (intron)
4: H2B-mCherry-CRISPR-Tag_v2 (6x) in intron
5: H2B-mCherry-CRISPR-Tag_v2 (4x) in intron

1: mCherry-LMNA
2: mCherry-LMNA-CRISPR-Tag_v1 in UTR
3: mCherry (intron)-LMNA
4: mCherry-LMNA-CRISPR-Tag_v2 (6x) in intron
5: mCherry-LMNA-CRISPR-Tag_v2 (4x) in intron

Supplementary Figure 12

The insertions of CRISPR-Tag have no significant effect on gene transcription (Related to Fig. 2).

Quantitative PCR measurement of transcript abundance of H2B (Left) and LMNA (right). The y axis shows transcript abundance values that are normalized to control cells (dCas9-GFP$_{14X}$ stable cell line), which does not have genomic modifications of target gene. RNA abundance is normalized to UBC. The data is displayed as means ± SEM for three technical replicates. One-way ANOVA analysis using PRISM does not show significant difference in relative RNA expression for all the comparisons.

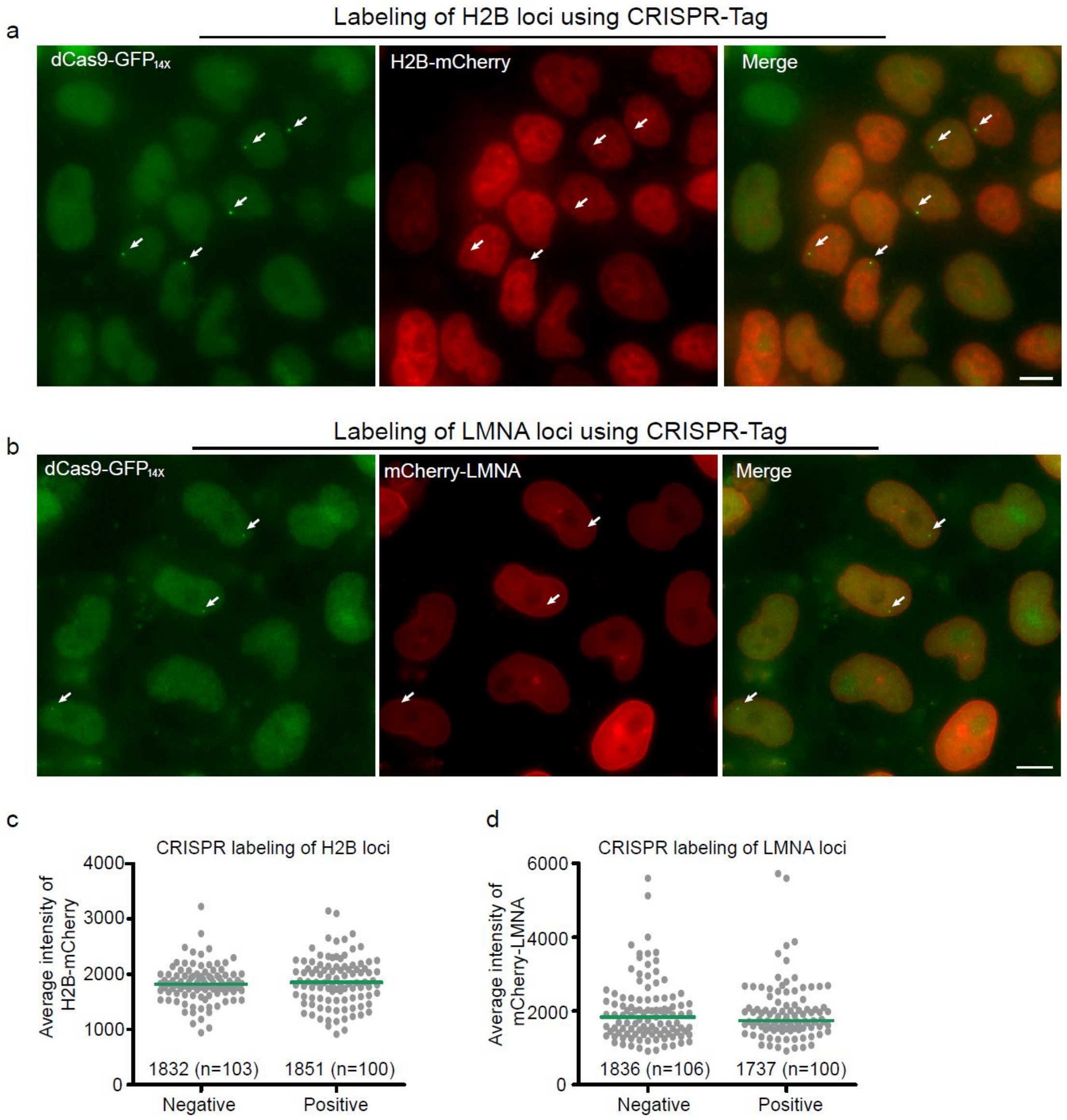

Supplementary Figure 13

Labeling protein-coding genes using CRISPR-Tag has no effect on protein expression (Related to Fig. 3).

(a, b) Representative images of H2B (a) and LMNA loci (b) labeled via CRISPR-Tags. Arrows indicate CRISPR labeling positive cells. Images in (a) are maximum intensity projections from z stacks, while images in (b) are from a single focal plane. Scale bars: 10 µm. (c, d) Comparisons of protein expression level in cells with and without CRISPR labeling. Each dot represents a single cell. Green line indicates the median value, n ≥ 100 cells.

Supplemental Table 1. CRISPR targeting sequences from *C. elegans*

Target gene	target sequence	PAM	sgRNA name	sgRNA design tool score	Efficiency (100%)	Reference
fox-1	GATATCGTTTACCAAAACGG	GGG	sgTS1	86	21	Farboud and Meyer, 2015
avr-14	GATTGGAGAGTTAGACCACG	TGG	sgTS2	86	20	Kim *et al.*, 2014
Y62E10A.17	GCACCGATGCTCTCCGAGG	AGG	sgTS3	85	57	Farboud and Meyer, 2015
klp-12	GATCCACAAGTTACAATTGG	TGG	sgTS4	77	80.3	Friedland *et al.*, 2013
C35E7.6	GGGCACCATACCGAGTGATG	GGG	sgTS5	90	100	Kim *et al.*, 2014
dpy-10	GCTACCATAGGCACCACGAG	CGG	sgTS6	87	High	Mouridi *et al.*, 2017
Artificial	GAGTAGTCAGAATAAGCGG	AGG	sgTS7	89	-	-

sgRNA activity was predicted by the web tool (http://crispr.mit.edu/). Editing efficiency was characterized by previous studies in *C. elegans*.

Supplemental Table 2. HDR-based knock-in efficiency

Target gene	Knock-in fragment	Donor plasmid	knock-in position (CRISPR-Tag position)	Cell type	Efficiency
H2B	mCherry	Double-cut	N-terminal	293T	18.1%
H2B	mCherry-CRISPR-Tag_v1	Double-cut	N-terminal (UTR)	293T	10.7%
H2B	mCherry	Regular	N-terminal	HeLa	0.3%
H2B	mCherry	Double-cut	N-terminal	HeLa	1.9%
H2B	mCherry-CRISPR-Tag_v1(6x)	Double-cut	N-terminal (UTR)	HeLa	0.8%
H2B	mCherry-CRISPR-Tag_v1(5x)	Double-cut	N-terminal (UTR)	HeLa	0.3%
H2B	mCherry-CRISPR-Tag_v1(3x)	Double-cut	N-terminal (UTR)	HeLa	1.1%
H2B	mCherry-CRISPR-Tag_v1(2x)	Double-cut	N-terminal (UTR)	HeLa	0.6%
H2B	mCherry-CRISPR-Tag_v1(1x)	Double-cut	N-terminal (UTR)	HeLa	0.8%
H2B	mCherry-CRISPR-Tag (7 bp)	Double-cut	N-terminal (UTR)	HeLa	0.3%
H2B	mCherry-CRISPR-Tag (37 bp)	Double-cut	N-terminal (UTR)	HeLa	0.3%
H2B	mCherry-CRISPR-Tag (52 bp)	Double-cut	N-terminal (UTR)	HeLa	0.3%
H2B	mCherry-intron	Double-cut	N-terminal	HeLa	0.8%
H2B	mCherry-CRISPR-Tag_v2 (6x)	Double-cut	N-terminal (intron)	HeLa	0.1%
H2B	mCherry-CRISPR-Tag_v2 (4x)	Double-cut	N-terminal (intron)	HeLa	0.4%
LMNA	mCherry	Double-cut	C-terminal	HeLa	0.6%
LMNA	mCherry-CRISPR-Tag_v1(6x)	Double-cut	C-terminal (UTR)	HeLa	0.1%
LMNA	mCherry-intron	Double-cut	C-terminal	HeLa	0.6%
LMNA	mCherry-CRISPR-Tag_v2 (6x)	Double-cut	C-terminal (intron)	HeLa	0.6%
LMNA	mCherry-CRISPR-Tag_v2 (4x)	Double-cut	C-terminal (intron)	HeLa	1.2%
HSPA8	mCherry	Double-cut	N-terminal	HeLa	0.4%
HSPA8	mCherry-CRISPR-Tag_v1(6x)	Double-cut	N-terminal (UTR)	HeLa	0.2%

Supplemental Table 3. DNA sequence of dCas9-GFP11$_{14X}$

Sequence (5' to 3')
GCCACCATGCCCAAGAAGAAGCGCAAGGTGGGACGCGTCTGCAGGATATCAAGCTTGCGGTACCGCGGGC<br>CCGGGATCGCCACCATGGACAAGAAGTACAGCATCGGCCTGGCCATCGGCACCAACTCTGTGGGCTGGGCC<br>GTGATCACCGACGAGTACAAGGTGCCCAGCAAGAAATTCAAGGTGCTGGGCAACACCGACCGGCACAGCA<br>TCAAGAAGAACCTGATCGGCGCCCTGCTGTTCGACAGCGGAGAAACAGCCGAGGCCACCCGGCTGAAGA<br>GAACCGCCAGAAGAAGATACACCAGACGGAAGAACCGGATCTGCTATCTGCAAGAGATCTTCAGCAACGA<br>GATGGCCAAGGTGGACGACAGCTTCTTCCACAGACTGGAAGAGTCCTTCCTGGTGGAAGAGGATAAGAAG<br>CACGAGCGGCACCCCATCTTCGGCAACATCGTGGACGAGGTGGCCTACCACGAGAAGTACCCCACCATCTA<br>CCACCTGAGAAAGAAACTGGTGGACAGCACCGACAAGGCCGACCTGCGGCTGATCTATCTGGCCCTGGCC<br>CACATGATCAAGTTCCGGGGCCACTTCCTGATCGAGGGCGACCTGAACCCCGACAACAGCGACGTGGACA<br>AGCTGTTCATCCAGCTGGTGCAGACCTACAACCAGCTGTTCGAGGAAAACCCCATCAACGCCAGCGGCGTG<br>GACGCCAAGGCCATCCTGTCTGCCAGACTGAGCAAGAGCAGACGGCTGGAAAATCTGATCGCCCAGCTGC<br>CCGGCGAGAAGAAGAATGGCCTGTTCGGCAACCTGATTGCCCTGAGCCTGGGCCTGACCCCCAACTTCAA<br>GAGCAACTTCGACCTGGCCGAGGATGCCAAACTGCAGCTGAGCAAGGACACCTACGACGACGACCTGGAC<br>AACCTGCTGGCCCAGATCGGCGACCAGTACGCCGACCTGTTTCTGGCCGCCAAGAACCTGTCCGACGCCAT<br>CCTGCTGAGCGACATCCTGAGAGTGAACACCGAGATCACCAAGGCCCCCCTGAGCGCCTCTATGATCAAGA<br>GATACGACGAGCACCACCAGGACCTGACCCTGCTGAAAGCTCTCGTGCGGCAGCAGCTGCCTGAGAAGTA<br>CAAAGAGATTTTCTTCGACCAGAGCAAGAACGGCTACGCCGGCTACATCGATGGCGGAGCCAGCCAGGAA<br>GAGTTCTACAAGTTCATCAAGCCCATCCTGGAAAAGATGGACGGCACCGAGGAACTGCTCGTGAAGCTGAA<br>CAGAGAGGACCTGCTGCGGAAGCAGCGGACCTTCGACAACGGCAGCATCCCCCACCAGATCCACCTGGGA<br>GAGCTGCACGCCATTCTGCGGCGGCAGGAAGATTTTTACCCATTCCTGAAGGACAACCGGGAAAAGATCG<br>AGAAGATCCTGACCTTCCGCATCCCCTACTACGTGGGCCCTCTGGCCAGGGGAAACAGCAGATTCGCCTGG<br>ATGACCAGAAAGAGCGAGGAAACCATCACCCCCTGGAACTTCGAGGAAGTGGTGGACAAGGGCGCCAGC<br>GCCCAGAGCTTCATCGAGCGGATGACCAACTTCGATAAGAACCTGCCCAACGAGAAGGTGCTGCCCAAGCA<br>CAGCCTGCTGTACGAGTACTTCACCGTGTACAACGAGCTGACCAAAGTGAAATACGTGACCGAGGGAATGA<br>GAAAGCCCGCCTTCCTGAGCGGCGAGCAGAAAAAAGCCATCGTGGACCTGCTGTTCAAGACCAACCGGAA<br>AGTGACCGTGAAGCAGCTGAAAGAGGACTACTTCAAGAAAATCGAGTGCTTCGACTCCGTGGAAATCTCCG<br>GCGTGGAAGATCGGTTCAACGCCTCCCTGGGCACATACCACGATCTGCTGAAAATTATCAAGGACAAGGAC<br>TTCCTGGACAATGAGGAAAACGAGGACATTCTGGAAGATATCGTGCTGACCCTGACACTGTTTGAGGACAG<br>AGAGATGATCGAGGAACGGCTGAAAACCTATGCCCACCTGTTCGACGACAAAGTGATGAAGCAGCTGAAG<br>CGGCGGAGATACACCGGCTGGGGCAGGCTGAGCCGGAAGCTGATCAACGGCATCCGGGACAAGCAGTCC<br>GGCAAGACAATCCTGGATTTCCTGAAGTCCGACGGCTTCGCCAACAGAAACTTCATGCAGCTGATCCACGA<br>CGACAGCCTGACCTTTAAAGAGGACATCCAGAAAGCCCAGGTGTCCGGCCAGGGCGATAGCCTGCACGAG<br>CACATTGCCAATCTGGCCGGCAGCCCCGCCATTAAGAAGGGCATCCTGCAGACAGTGAAGGTGGTGGACG<br>AGCTCGTGAAAGTGATGGGCCGGCACAAGCCCGAGAACATCGTGATCGAAATGGCCAGAGAGAACCAGAC<br>CACCCAGAAGGGACAGAAGAACAGCCGCGAGAGAATGAAGCGGATCGAAGAGGGCATCAAAGAGCTGG<br>GCAGCCAGATCCTGAAAGAACACCCCGTGGAAAACACCCAGCTGCAGAACGAGAAGCTGTACCTGTACTAC<br>CTGCAGAATGGGCGGGATATGTACGTGGACCAGGAACTGGACATCAACCGGCTGTCCGACTACGATGTGGA<br>CGCTATCGTGCCTCAGAGCTTTCTGAAGGACGACTCCATCGATAACAAAGTGCTGACTCGGAGCGACAAGA<br>ACCGGGGCAAGAGCGACAACGTGCCCTCCGAAGAGGTCGTGAAGAAGATGAAGAACTACTGGCGCCAGC<br>TGCTGAATGCCAAGCTGATTACCCAGAGGAAGTTCGACAATCTGACCAAGGCCGAGAGAGGCGGCCTGAG<br>CGAACTGGATAAGGCCGGCTTCATCAAGAGACAGCTGGTGGAAACCCGGCAGATCACAAAGCACGTGGCA<br>CAGATCCTGGACTCCCGGATGAACACTAAGTACGACGAGAACGACAAACTGATCCGGGAAGTGAAAGTGAT<br>CACCCTGAAGTCCAAGCTGGTGTCCGATTTCCGGAAGGATTTCCAGTTTTACAAAGTGCGCGAGATCAACA<br>ACTACCACCACGCCCACGACGCCTACCTGAACGCCGTCGTGGGAACCGCCCTGATCAAAAAGTACCCTAAG<br>CTGGAAAGCGAGTTCGTGTACGGCGACTACAAGGTGTACGACGTGCGGAAGATGATCGCCAAGAGCGAGC<br>AGGAAATCGGCAAGGCTACCGCCAAGTACTTCTTCTACAGCAACATCATGAACTTTTTCAAGACCGAGATTA<br>CCCTGGCCAACGGCGAGATCCGGAAGCGGCCTCTGATCGAGACAAACGGCGAAACAGGCGAGATCGTGTG<br>GGATAAGGGCCGGGACTTTGCCACCGTGCGGAAAGTGCTGTCTATGCCCCAAGTGAATATCGTGAAAAAGA<br>CCGAGGTGCAGACAGGCGGCTTCAGCAAAGAGTCTATCCTGCCCAAGAGGAACAGCGACAAGCTGATCGC<br>CAGAAAGAAGGACTGGGACCCTAAGAAGTACGGCGGCTTCGACAGCCCCACCGTGGCCTATTCTGTGCTG<br>GTGGTGGCCAAAGTGGAAAAGGGCAAGTCCAAGAAACTGAAGAGTGTGAAAGAGCTGCTGGGGATCACC<br>ATCATGGAAAGAAGCAGCTTCGAGAAGAATCCCATCGACTTTCTGGAAGCCAAGGGCTACAAAGAAGTGA<br>AAAAGGACCTGATCATCAAGCTGCCTAAGTACTCCCTGTTCGAGCTGGAAAACGGCCGGAAGAGAATGCTG

GCCTCTGCCGGCGAACTGCAGAAGGGAAACGAACTGGCCCTGCCCTCCAAATATGTGAACTTCCTGTACCT
GGCCAGCCACTATGAGAAGCTGAAGGGCTCCCCCGAGGATAATGAGCAGAAACAGCTGTTTGTGGAACAG
CACAAACACTACCTGGACGAGATCATCGAGCAGATCAGCGAGTTCTCCAAGAGAGTGATCCTGGCCGACGC
TAATCTGGACAAGGTGCTGAGCGCCTACAACAAGCACAGAGACAAGCCTATCAGAGAGCAGGCCGAGAAT
ATCATCCACCTGTTTACCCTGACCAATCTGGGAGCCCCTGCCGCCTTCAAGTACTTTGACACCACCATCGACC
GGAAGAGGTACACCAGCACCAAAGAGGTGCTGGACGCCACCCTGATCCACCAGAGCATCACCGGCCTGTA
CGAGACACGGATCGACCTGTCTCAGCTGGGAGGCGACGCCTATCCCTATGACGTGCCCGATTATGCCAGCCT
GGGCAGCGGCTCCCCCAAGAAAAAACGCAAGGTGGAAGATCCTAAGAAAAAGCGGAAAGTGGACGGCAT
TGGTAGTGGGAGCAACGGCAGCAGCGGATCCATGCGGGACCACATGGTGCTGCATGAATACGTGAACGCG
GCGGGGATAACGGGTGGGTCCGGAGGTAGAGACCATATGGTTCTCCACGAGTACGTCAACGCAGCGGGCA
TTACAGGCGGTTCAGGTGGCAGGGATCACATGGTGTTACATGAATATGTAAATGCGGCTGGAATCACCGGA
GGATCTGGAGGCCGGGACCATATGGTCTTGCACGAGTACGTTAACGCTGCTGGTATAACCGGGGGCTCGGG
CGGTAGAGATCACATGGTACTCCATGAGTATGTCAATGCCGCGGGTATAACTGGTGGGTCAGGTGGACGCG
ACCATATGGTTTTACATGAATATGTGAACGCAGCAGGAATTACTGGCGGTTCTGGGGGTAGGGATCATATGG
TACTACACGAGTACGTGAACGCCGCTGGCATCACCGGCGGATCAGGGGGCCGCGACCATATGGTTCTCCAC
GAATACGTGAATGCCGCCGGAATTACAGGCGGATCGGGAGGTCGTGATCACATGGTACTACATGAGTACGTA
AACGCGGCGGGGATCACGGGAGGATCTGGTGGTCGAGACCATATGGTGCTTCACGAGTATGTTAACGCAGC
AGGTATTACCGGCGGTTCTGGTGGCAGAGACCATATGGTCCTGCATGAGTATGTCAATGCCGCTGGCATCAC
TGGAGGCTCAGGTGGAAGAGATCACATGGTTCTACACGAATATGTGAATGCAGCCGGTATCACTGGTGGGT
CAGGAGGGCGCGACCACATGGTACTCCATGAATACGTTAATGCAGCTGGGATTACAGGGGGTTCCGGCGGT
CGGGATCATATGGTTTTGCATGAGTACGTCAACGCTGCCGGAATCACACAGCGGCCGCAAGGTGGAGGTGG
ACCCAAGAAGAAGCGCAAGGTGGGAAGCGGAGCTACTAACTTCAGCCTGCTGAAGCAGGCTGGAGACGT
GGAGGAGAACCCTGGACCTGCACCAGGAAGTATGAGCGAGCTGATTAAGGAGAACATGCACATGAAGCTG
TACATGGAGGGCACCGTGGACAACCATCACTTCAAGTGCACATCCGAGGGCGAAGGCAAGCCCTACGAGG
GCACCCAGACCATGAGAATCAAGGTGGTCGAGGGCGGCCCTCTCCCCTTCGCCTTCGACATCCTGGCTACTA
GCTTCCTCTACGGCAGCAAGACCTTCATCAACCACACCCAGGGCATCCCCGACTTCTTCAAGCAGTCCTTCC
CTGAGGGCTTCACATGGGAGAGAGTCACCACATACGAAGACGGGGGCGTGCTGACCGCTACCCAGGACAC
CAGCCTCCAGGACGGCTGCCTCATCTACAACGTCAAGATCAGAGGGGTGAACTTCACATCCAACGGCCCTG
TGATGCAGAAGAAAACACTCGGCTGGGAGGCCTTCACCGAGACGCTGTACCCCGCTGACGGCGGCCTGGA
AGGCAGAAACGACATGGCCCTGAAGCTCGTGGGCGGGAGCCATCTGATCGCAAACATCAAGACCACATATA
GATCCAAGAAACCCGCTAAGAACCTCAAGATGCCTGGCGTCTACTATGTGGACTACAGACTGGAAAGAATC
AAGGAGGCCAACAACGAGACCTACGTCGAGCAGCACGAGGTGGCAGTGGCCAGATACTGCGACCTCCCTA
GCAAACTGGGGCACAAGCTTAATGGTGGAGGTCCAAAAAAGAAAAGGAAGGTCTAA

(Red text is nuclear localization signal peptide (NLS); orange text is nuclease-deficient Cas9 (dCas9); test marked by yellow is 14 copies of GFP11; underlined text is P2A sequence; blue text is BFP.)

Supplementary Table 4. DNA sequence of GFP1-10

Sequence (5' to 3')
GCCACCATGTCCAAAGGAGAAGAACTGTTTACCGGTGTTGTGCCAATTTTGGTTGAACTCGATGGTGATGTC<br>AACGGACATAAGTTCTCAGTGAGAGGCGAAGGAGAAGGTGACGCCACCATTGGAAAATTGACTCTTAAATT<br>CATCTGTACTACTGGTAAACTTCCTGTACCATGGCCGACTCTCGTAACAACGCTTACGTACGGAGTTCAGTGC<br>TTTTCGAGATACCCAGACCATATGAAAAGACATGACTTTTTTAAGTCGGCTATGCCTGAAGGTTACGTGCAA<br>GAAAGAACAATTTCGTTCAAAGATGATGGAAAATATAAAACTAGAGCAGTTGTTAAATTTGAAGGAGATACT<br>TTGGTTAACCGCATTGAACTGAAAGGAACAGATTTTAAAGAAGATGGTAATATTCTTGGACACAAACTCGAA<br>TACAATTTTAATAGTCATAACGTATACATCACTGCTGATAAGCAAAAGAACGGAATTAAAGCGAATTTCACAGT<br>ACGCCATAATGTAGAAGATGGCAGTGTTCAACTTGCCGACCATTACCAACAAAACACCCCTATTGGAGACGG<br>TCCGGTACTTCTTCCTGATAATCACTACCTCTCAACACAAACAGTCCTGAGCAAAGATCCAAATGAAAAAGG<br>AACAGGTGGCGGCGGAAGTCCCAAGAAGAAGCGCAAGGTGTAA
(Red text is nuclear localization signal peptide (NLS); green text is GFP1-10)

Supplemental Table 5. sgRNA guide sequences to target human genome

Target	target sequence	PAM	Assay
H2B	GCGAGCGCCAGGTCCCGGCA	GGG	DNA eding
LMNA	GCTTGTCTCCCTCTACAGCC	TGG	DNA editing
HSPA8	GCTACATCTACACTTGGT	TGG	DNA editing
5S_rDNA	GGCCTGGTTAGTACTTGGAT	TGG	DNA labeling
MUC4(repetitive)	GTGGCGTGACCTGTGGATGCTG	AGG	DNA labeling
MUC4 (non-repetitive)	GCTCACTGCAACCTCCACCTCCC	AGG	DNA labeling
	GACAGAGTCTCGCTCTCTCTCCC	AGG	
	GAAGAGGAGAAAAGTGGGGAAG	AGG	
	GAACAGAGGGCCAGAGAGCAGCC	CGG	
	GTCTTTCTCTCTGCGAGTAAGCCT	AGG	
	GTACACCCTTGTGTACAGAGCT	GGG	
	GAAAACTCATGTAAAGCTGCA	GGG	
	GGAGGCGGCCAGGGCGCAGA	GGG	
	GCTTTTAAACCCGAGCTCAG	AGG	
	GTAGCCCCGGCATTGGCCTT	GGG	
	GCCTGTGGGAGATGTTCCCTC	GGG	
	GAGTCTTTGGGGGAGAGTCT	GGG	
	GCTCCTGCCCTGCCTCTCAGC	AGG	
	GGCTGCAAGAGAAGCCATGC	TGG	
	GATGTTTCAGGACTAGGCTGA	GGG	
	GCTGGAGGGTGGGGAGGTGTA	GGG	
	GCATATTTGAGGAGCTTCCT	GGG	
	GGTGGGATGAGCACTGGAGC	GGG	
	GCCCTGCAGATGTGGTTGA	AGG	
	GAGGCTGGGGCTTGGGGCGCC	GGG	
	GTCTTTGCCGTGAACTGTTC	TGG	
	GACCGGGGCCCTGGGGAGACAC	GGG	
	GCTGGACACTCAGCTCCATG	TGG	
	GAGCGCAGAGGGGCAAGACCT	GGG	
	GAGCTGGGCCAGGAGAGGAGA	TGG	
	GTTTCCTTAAGGAACAGCCC	TGG	
	GAGAAGGAGTGAAGGACTGT	TGG	
	GCTCCACGACATGCCTAGCTTCTTC	GGG	
	GACCGGGCATGACCAGGGCCT	TGG	
	GTTCCTTTTGGCTCCCTGAAG	GGG	
	GGGTCTGTTTGCACACTTGC	CGG	
	GCCCAGGCCAGAGGAAAAACACA	GGG	
	GCAGACAGAGGTGGGCTAGACA	AGG	
	GCCCCAGGCAGGAATGACTCAGA	AGG	
	GACCCAGTTGCCTTTCCCTG	TGG	
	GCCACAGCGCACTCCACGGGGAA	GGG	

Supplementary Table 6. DNA sequence of CRISPR-Tag

Version 1 (CRISPR-Tag_v1):

Sequence (5' to 3')
TGACGATCCACAAGTTACAATTGGTGGCGACAAGGATATCGTTTACCAAAACGGGGGCATCAGCCGCACCGATGCTCTCCGAGGAGGCACCTACGATTGGAGAGTTAGACCACGTGGgggctaattcactcccaacgaGCAAGATCCACAAGTTACAATTGGTGGCGACAAGGATATCGTTTACCAAAACGGGGGCATCAGCCGCACCGATGCTCTCCGAGGAGGCACCTACGATTGGAGAGTTAGACCACGTGGccttgatctgtggatctaccaGAACGATCCACAAGTTACAATTGGTGGCGACAAGGATATCGTTTACCAAAACGGGGGCATCAGCCGCACCGATGCTCTCCGAGGAGGCACCTACGATTGGAGAGTTAGACCACGTGGggcagaactacacaccagggcCAGTGATCCACAAGTTACAATTGGTGGCGACAAGGATATCGTTTACCAAAACGGGGGCATCAGCCGCACCGATGCTCTCCGAGGAGGCACCTACGATTGGAGAGTTAGACCACGTGGcatcggcatctttgacgcaagACACGATCCACAAGTTACAATTGGTGGCGACAAGGATATCGTTTACCAAAACGGGGGCATCAGCCGCACCGATGCTCTCCGAGGAGGCACCTACGATTGGAGAGTTAGACCACGTGGccgcttgttacaccctgtgagTGTCGATCCACAAGTTACAATTGGTGGCGACAAGGATATCGTTTACCAAAACGGGGGCATCAGCCGCACCGATGCTCTCCGAGGAGGCACCTACGATTGGAGAGTTAGACCACGTGGGCAGTAGTATCTTTGCAAGTGACTC<br><br>(Underlined text is repetitive elements; red text is the four nucleotides left by Golden Gate assembly; text marked by grey is TS1 sequence; text marked highlighted in yellow is TS2 sequence; text marked by blue is TS3 sequence; text marked by green is TS4 sequence; text in lower case is unique sequences between two neighboring repetitive elements.)

Version 2 (CRISPR-Tag_v2):

Sequence (5' to 3')
TGACGAGTAGTCAGAATAAGCGGAGGCCCTGAGGGGCACCATACCGAGTGATGGGGCCCATCAGCTACCATAGGCACCACGAGCGGCAGggcagaactacacaccagggcCAGTGAGTAGTCAGAATAAGCGGAGGCCCTGAGGGGCACCATACCGAGTGATGGGGCCCATCAGCTACCATAGGCACCACGAGCGGCAGcatcggcatctttgacgcaagACACGAGTAGTCAGAATAAGCGGAGGCCCTGAGGGGCACCATACCGAGTGATGGGGCCCATCAGCTACCATAGGCACCACGAGCGGCAGccgcttgttacaccctgtgagTGTCGAGTAGTCAGAATAAGCGGAGGCCCTGAGGGGCACCATACCGAGTGATGGGGCCCATCAGCTACCATAGGCACCACGAGCGGCAGGCAGTAGTATCTTTGCAAGTGACTC<br><br>(Underlined text is repetitive elements; red text is the four nucleotides left by Golden Gate assembly; text marked by magenta is TS5 sequence; text highlighted in red is TS6 sequence; text marked by dark green is TS7 sequence; text in lower case is unique sequences between two neighboring repetitive elements.)

Supplementary Table 7. DNA sequence of mCherry fusions with intron and CRISPR-Tag

mCherry with intron:

Sequence (5' to 3')
ATGGTGAGCAAGGGCGAGGAGGATAACATGGCCATCATCAAGGAGTTCATGCGCTTCAAGGTGCACATGG<br>AGGGCTCCGTGAACGGCCACGAGTTCGAGATCGAGGGCGAGGGCGAGGGCCGCCCCTACGAGGGCACCC<br>AGACCGCCAAGCTGAAGGTGACCAAGGGTGGCCCCCTGCCCTTCGCCTGGGACATCCTGTCCCCTCAGTTC<br>ATGTACGGCTCCAAGGCCTACGTGAAGCACCCCGCCGACATCCCCGACTACTTGAAGCTGTCCTTCCCCGAG<br>GGCTTCAAGTGGGAGCGCGTGATGAACTTCGAGGACGGCGGCGTGGTGACCGTGACCCAGGACTCCTCCC<br>TGCAGGACGGCGAGTTCATCTACAAGGTGAAGCTGCGCGGCACCAACTTCCCCTCCGACGGCCCCGTAATG<br>CAGAAGAAGACCATGGGCTGGGAGGCCTCCTCCGAGCGGATGTACCCCGAGGACGGCGCCCTGAAGGGC<br>GAGATCAAGCAGAGGCTGAAGCTGAAGGACGGCGGCCACTACGACGCTGAGGTCAAGACCACCTACAAG<br>GCCAAGAAGCCCGTGCAGCTGCCCGGCGCCGTAAGTATGAAATTCAGGGATACGGccaccttgttggCATATTT<br>GCCAAATAGTGGAAATGTGAAGTACTGACAAAACTTTTCCCTTTTTCAATCTAATAGTACAACGTCAACATCA<br>AGTTGGACATCACCTCCCACAACGAGGACTACACCATCGTGGAACAGTACGAACGCGCCGAGGGCCGCCA<br>CTCCACCGGCGGCATGGA CGAGCTGTACAAGTAA
(Red text is the coding sequence of mCherry; text highlighted in yellow is the intron from human HSPA5 gene with small modifications; underlined text in lower case is BstXI restriction site.)

mCherry with intron and CRISPR-Tag_v2:

Sequence (5' to 3')
ATGGTGAGCAAGGGCGAGGAGGATAACATGGCCATCATCAAGGAGTTCATGCGCTTCAAGGTGCAC<br>ATGGAGGGCTCCGTGAACGGCCACGAGTTCGAGATCGAGGGCGAGGGCGAGGGCCGCCCCTACG<br>AGGGCACCCAGACCGCCAAGCTGAAGGTGACCAAGGGTGGCCCCCTGCCCTTCGCCTGGGACAT<br>CCTGTCCCCTCAGTTCATGTACGGCTCCAAGGCCTACGTGAAGCACCCCGCCGACATCCCCGACTA<br>CTTGAAGCTGTCCTTCCCCGAGGGCTTCAAGTGGGAGCGCGTGATGAACTTCGAGGACGGCGGC<br>GTGGTGACCGTGACCCAGGACTCCTCCCTGCAGGACGGCGAGTTCATCTACAAGGTGAAGCTGCG<br>CGGCACCAACTTCCCCTCCGACGGCCCCGTAATGCAGAAGAAGACCATGGGCTGGGAGGCCTCCT<br>CCGAGCGGATGTACCCCGAGGACGGCGCCCTGAAGGGCGAGATCAAGCAGAGGCTGAAGCTGAA<br>GGACGGCGGCCACTACGACGCTGAGGTCAAGACCACCTACAAGGCCAAGAAGCCCGTGCAGCTG<br>CCCGGCGCCgtaagtatgaaattcagggatacggccaccttgttggcgggccagatatacgcgttgacGAGTAGTCAGAATAAG<br>CGGAGGCCCTGAGGGGCACCATACCGAGTGATGGGGCCCATCAGCTACCATAGGCACCACGAGCG<br>GCAGGGCAGAACTACACACCAGGGCCAGTGAGTAGTCAGAATAAGCGGAGGCCCTGAGGGGCAC<br>CATACCGAGTGATGGGGCCCATCAGCTACCATAGGCACCACGAGCGGCAGCATCGGCATCTTTGAC<br>GCAAGACACGAGTAGTCAGAATAAGCGGAGGCCCTGAGGGGCACCATACCGAGTGATGGGGCCCA<br>TCAGCTACCATAGGCACCACGAGCGGCAGCCGCTTGTTACACCCTGTGAGTGTCGAGTAGTCAGAA<br>TAAGCGGAGGCCCTGAGGGGCACCATACCGAGTGATGGGGCCCATCAGCTACCATAGGCACCACG<br>AGCGGCAGgcagtagtatctttgcaagtgactcacccaccttgttggcatatttgccaaatagtggaaatgtgaagtactgacaaaacttttccc<br>tttttcaatctaatagTACAACGTCAACATCAAGTTGGACATCACCTCCCACAACGAGGACTACACCATCGTG<br>GAACAGTACGAACGCGCCGAGGGCCGCCACTCCACCGGCGGCATGGACGAGCTGTACAAGTAA
(Red text is the coding sequence of mCherry; underlined text is the intron region harboring the CRISPR-Tag_v2 labeled by yellow.)

Supplementary Table 8. Primers sets used for qRT-PCR

Name of genes	Forward primer (5' to 3')	Backward primer (5' to 3')
H2B	AGCCGCAAAGAGAGCTACTCCA	CGCTCGAAGATGTCGTTGACGA
LMNA	CTGAGCACTGCTCTCAGTGAG	CAGCCTGTTCTCAGCATCCAC
UBC	CCCAGTATCAGCAGAAGGACA	ATCGCCGAGAAGGGACTACTT
