# Supplementary material for: Efficient labeling and imaging of protein-coding genes in living cells using CRISPR-Tag
Source: Nat Commun. 2018 Nov 29;9:5065. doi: 10.1038/s41467-018-07498-y (PMC6265289; doi:10.1038/s41467-018-07498-y)
Supplement: Supplementary file 5 — Description of Additional Supplementary Files [file 41467_2018_7498_MOESM5_ESM.docx]

**Title:** Supplementary Movie 1
**Description:** Live cell imaging of H2B-mCherry (red) and H2B loci labeled by dCas9-GFP14X (green) using the CRISPR-Tag system (CRISPR-Tag_v1, 803 bp). 4 hr movie is shown. Z projection with merge signal is shown. Scale bar: 10 µm.

**Title:** Supplementary Movie 2
**Description:** Live cell imaging LMNA loci labeled by dCas9-GFP14X (green) using the CRISPR-Tag system (CRISPR-Tag_v2, 635 bp). 30 min movie is shown. Images were acquired from a single plane with 1-second interval. Scale bar: 20 µm.

**Title:** Supplementary Movie 3
**Description:** Live cell imaging of mCherry-LMNA (red) and LMNA loci labeled by dCas9-GFP14X (green) using the CRISPR-Tag system (CRISPR-Tag_v2, 635 bp). 6 hr movie is shown. Z projection with merge signal is shown. Scale bar: 7 µm.
